# Multi-Dimensional Dynamics of Psychological Health Disparities under the COVID-19 in Japan: Fairness/Justice in Socio-Economic and Ethico-Political Factors

**DOI:** 10.3390/ijerph192416437

**Published:** 2022-12-08

**Authors:** Masaya Kobayashi, Hikari Ishido, Jiro Mizushima, Hirotaka Ishikawa

**Affiliations:** 1 Graduate School of Social Sciences, Chiba University, Chiba 263-8522, Japan; 2 Graduate School of Global and Transdisciplinary Studies, Chiba University, Chiba 263-8522, Japan; 3 Graduate School of Humanities and Studies on Public Affairs, Chiba University, Chiba 263-8522, Japan

**Keywords:** well-being, psychological health, socio-economic factors, positive psychology, fairness, justice

## Abstract

This article addresses citizens’ psychological health disparities in pandemic-stricken Japan from the perspective of positive psychology with a collective/political perspective. Our analysis of three internet surveys in 2020 and 2021 in Japan indicates most people’s well-being declined continuously during this period, while some people’s well-being increased. As previous studies of health inequality proved about physical health, the objective income/assets level has influenced psychological inequality. This paper demonstrated this relation in Japan, although it is often mentioned as an egalitarian country with comparatively better health conditions. Moreover, psychological levels and changes have been associated with biological, natural environmental, cultural, and social factors. Social factors include economic, societal-community, and political factors, such as income/assets, stratification, general trust, and fairness/justice. Accordingly, multi-dimensional disparities are related to psychological health disparity; tackling the disparities along the multi-layered strata is desirable. Furthermore, subjective perception of fairness/justice is significantly associated with the level of psychological health and mitigating its decrease. Thus, fairness and justice are found to be dynamic and protective factors against the decline of psychological health. While relatively little literature on health inequality analyzes fairness/justice philosophically, this paper highlights these together with income/assets by clarifying the significance of multi-dimensional factors: natural environmental, cultural, socioeconomic, and political.

## 1. Introduction

As the problem of COVID-19 continues on a global scale from 2019 to the present, it is generally thought that people’s mental state in Japan has deteriorated. This change is due to emergency declarations and restrictions on behavior and outings, with effects such as deterioration of living conditions and increased suicides.

Until now, most studies in psychology and psychiatry have focused their exploration on mental diseases to explore the necessary condition of psychological health. Nevertheless, the concept of “psychological health” can include not only the absence of illness but also a better state of being. As one of the articles in the first Special Issue, “Survey about Psychological Health”, in this journal mentioned [1], positive psychology has rapidly studied this better psychological state in recent years [2,3,4,5].

So then, this paper investigates psychological health from this perspective and examines its conditions. While psychological health would play a central role in this paper, as is often the case with psychology as usual, the term ‘psychological health’ tended to have been used in the context of negative phenomena such as psychological disease, disorder, and medicine. In contrast, this study uses the word with both positive and negative aspects.

Moreover, The Special Issue “Survey about Psychological Health” defined psychological health as “the development of an individual’s optimal state of mind within the limits of maintaining physical, mental, and emotional compatibility with others”. Accordingly, this notion is a social psychological concept that refers to not only the individual but also the social aspect. 

Many studies have focused on individual psychology to investigate well-being (hereafter WB) in positive psychology. Nevertheless, several studies have focused on social or political dimensions [6,7,8,9,10]. In line with these, in recent years, new studies have also called attention to the influence of the collective dimension, and the previous papers concerning this article have proposed a new research area called positive political psychology [11,12]. 

Based on this perspective, introducing a positive and social perspective into the prevalent approach to psychological health, this researches both the individual and social dimensions concerning both negative and positive human psychology. 

In doing so, we would like to focus on the COVID-19 issue. While most studies, including several articles in this journal [13,14,15] and on positive psychology [7,16,17,18,19], have already examined this urgent worldwide problem from a psychological perspective, it is highly likely that social factors, including local anti-corona measures, are also significant factors in psychological change. In the discussion of health regarding the social dimension, the study of the health gap is essential, and the gap has been proved to be caused by social factors as well as biological factors. 

In the political science and economics fields in which the authors specialize, the relationship between inequality and fairness/justice is one of the most cardinal topics ([20,21]). Although there are merely a few psychological studies on fairness/justice [22,23,24], it may have an impact on individual WB. Accordingly, this paper analyzes the relationship between WB and the various factors from this perspective: it presents and discusses the results of a few detailed online surveys of Japanese citizens on WB in 2020 and 2021. 

Concomitant with the introduction of positive political psychology, the collaborative interdisciplinary framework between psychology and political philosophy has been pushed forward by the previous article concerning this paper ([11]). This study depends on the framework and analyzes the psychological gap empirically, referring to political philosophies. After examining the results, measures will be proposed to ameliorate psychological conditions’ deterioration or improvement.

## 2. Health Inequality/Disparity: Political Philosophy and Positive Psychology

### 2.1. Inequality and Justice in Political Philosophy

The relationship between inequality and justice is one of the most critical subjects in political philosophy, including libertarianism, liberalism, and communitarianism.

The first two philosophies are rights-based theories. Both libertarians and liberalists depend on their arguments of the concepts of individual rights, disregarding ethical views concerning the good life in judging justice in public policies. 

In particular, libertarians value liberty as much as possible, and they believe that government intervention in many areas on the ground of moral reasoning undermines liberty, such as abortion. Accordingly, they point out that governmental COVID-19 initiatives have undermined individual liberty.

On the other hand, liberals share this tendency concerning governmental moralistic interventions on personal actions, but their conception of rights differs from libertarians: while libertarians do not value welfare rights but property rights, liberals respect both rights. Then, the actual policy implications are different, especially regarding economic [25] and welfare policies. 

Consequently, libertarians believe inequalities that emerge from legitimate competition in a market economy are just, and the tax on the rich for welfare is unjust because it violates property rights. In contrast, egalitarian liberalists believe excessive inequalities are contrary to justice and redistribution is just because social or welfare rights exist and inequality violates them.

This argument is closely related to economic inequalities, such as income and assets. Libertarians believe inequalities are due to individual effort, ability, and ingenuity. In contrast, liberalists believe that social factors such as environment and social stratification are nonnegligible causes, and those inequalities for which individuals are not responsible should be corrected through social and political methods such as public policy. 

This point is concerned with the argument of personal responsibility for their various situations in luck egalitarianism, even among egalitarian liberalists ([25,26,27]). According to luck egalitarians on health, only when health inequalities reflect persons’ given luck inequalities are unjust; when they reflect their choices, these are not unjust. 

On the other hand, while libertarianism and liberalism are grounded solely on individual rights, communitarians value ethical ‘good life’ and communal moments as well as rights. They often argue that excessive inequality is contrary to justice through people’s deliberative arguments, often including ethical perspectives concerning the good life; it should be somehow reduced for the weak in the fellow citizens because this policy is regarded to contribute to the common good. This argument is based on ethical or moral reasoning rather than rights, but the conclusion regarding economic inequality is closer to egalitarian liberalism than libertarianism ([11,28,29,30]).

### 2.2. Health Inequality and Social Factors

What about health inequalities? Of course, inequalities exist because health varies significantly from person to person. Traditionally, this was attributed to the genetic constitution and smoking, foods, drinking, exercise, and other habits. If so, though the genetic constitution is fatal, the other factors are due to individual behavior; therefore, the individual is responsible for his or her health. If an individual makes an effort, the person can improve his or her health status, which is why awareness-raising and health education are essential. This conclusion is similar to the libertarian idea mentioned above.

However, this way of thinking is not sufficient. Some studies and reports have debunked the long-term effects of health education because their effects are less than expected ([31]; Chapter 12 in [32]). Moreover, valuable studies led by Richard G. Wilkinson and Michael Marmot revealed the amazing causality that the health gap (Marmot) results from inequality or an unfair society ([33,34,35,36,37,38,39]. These findings have facilitated studies on health inequality ([40,41]) and the ‘social determinants of health ([42]). 

Its core is the relative income hypothesis that the more egalitarian the distribution of income, the higher health, such as life expectancy; therefore, ‘almost every group in society (except perhaps the very rich) would reap the benefit of a more egalitarian distribution of income’ (p. xv, [43]). Moreover, Wilkinson proposed the psychosocial hypothesis: the reason for the health inequality lies in the psychosocial effect of low social status and poor quality of social relations, such as social capital [44], in hierarchical societies [43]. Despite some empirical or methodological criticisms, various counter-studies have made these theses remain convincing (pp. vii–xi, [43], pp. 35–39, [41]).

Based on the related empirical studies, international associations such as WHO and Healthy People enumerated the following factors: economic environment (economic stability), social environment (social and community context), educational environment (education access and quality), health services (health care access and quality), physical environment (neighborhood and built environment) [45,46,47]. 

Accordingly, this theme has become widely recognized [48,49,50]. So then, it is necessary not only for individuals to strive for good health but also to work on improving these social-psychological aspects. Just as liberalism and communitarianism consider economic equality to be an element of justice, the task of public policy on public health is to reduce health inequalities by improving social factors. Thus, discussions have approached the issue of justice and fairness in political philosophy [27,51].

Most arguments on health and justice have developed concerning liberalism represented by Rawls. While Rawls himself did not mention this issue, the simple idea is that health should be included in the lists of primary goods, as suggested by Kenneth Arrow [52]. 

An alternative theory was proposed by Norman Daniels, the most influential philosopher on this subject. He regarded health as having special moral importance instead of including it in primary goods. Then, he applied Rawls’ theory of justice to the health issue: first, the principle of fair equality of opportunity (the first part within Rawls’ second principle of justice) to the issue of health care in his *Just Health Care* [53], and secondly, the principle of fair equality opportunity and difference (both parts of the second principle) to the issue of health inequality in *Just Health: Meeting Health Needs Fairly* [54]. 

This development of his argument was inspired by the works on social determinants of health by two eminent Harvard public health researchers (Bruce Kennedy and Ichiro Kawachi). Accordingly, the three researchers published an influential article in their well-known provocative book: *Is Inequality Bad for Our Health?* [38]. They argued that greater socioeconomic inequality influences health inequality. As pathways, the socio-economic gradient can cause health inequality by educational inequality, undermining civil society, eroding social cohesion, reducing political participation, and undermining governmental responsiveness. In addition, they introduced Rawls’ theory of justice and argued that, on that ground, as a ‘striking result,’ ‘in a just society, health inequalities will be minimized, and population health status will be improved—in short, social justice is good for our health [38,55]’.

Similarly, from a communitarian perspective, since the realization of health is an essential component of the common good, the deliberative discussion would make policies to reduce health inequalities a justice imperative. Moreover, this political philosophy sheds light on the communal and ethical aspects, which libertarianism and liberalism dismiss.

This point is associated with the words concerning the health divide. Health inequality relates with general differences, which include both socio-economic gaps and unavoidable inequality caused by, for instance, age. Another similar concept of health disparity was proposed around 1990 in the United States: this term implies socio-economic and environmental disadvantages, such as ethnic groups and gender [56,57,58].

So then, Paula A. Braveman and others proposed a definition of ‘health disparities are systematic, plausibly avoidable health differences adversely affecting socially disadvantaged groups’: its opposite is health equity. This definition regards the dividing issue as justice represented by human rights [59,60,61] and implies an ethical and normative orientation that the disparity is ‘avoidable, unnecessary, and unjust’ (Margaret Whitehead) [62,63]. 

Accordingly, while this paper uses health inequality as a health difference in general, it defines a health disparity as the corresponding ethical concept of avoidable and unjust/unfair health inequality in general. Although its usage, especially in the United States, focuses on ethnicity and gender, this definition implies general ethical and normative issues concerning justice and fairness: it is a sub-issue of the broad problems of justice and fairness in society. Therefore, as this paper explores socio-economic and ethical elements, it will utilize not only health inequality but also health disparity/equity.

### 2.3. Positive Psychology and Justice on Psychological Health Disparity

The issue of health inequalities/disparities due to social factors has developed as a study of “social epidemiology” [64]. As a result, the “bio-psycho-social model” has been proposed in contrast to the conventional bio-medical model [32]. This new idea is the basis for “new public health”, emphasizing environmental factors (psychological, social, and material) [65,66,67].

This model has much in common with the discussion concerning WB in positive psychology. Lyubomirsky(p. 59, [68]) and Seligman (p. 45, [3]) have identified three factors as determinants of WB: genetic setpoints, circumstance, and intentional activities. Initially, the emphasis was on the genetic and individual intentional activity portions. However, Lyubomirsky [69] withdrew the numbers of the proportions concerning the three factors (50%, 10%, and 40%). Moreover, the importance of environmental factors, such as social inequality, has been recently highlighted. Similarly, heredity, individual effort, and environment influence physical health disparity. So then, this recognition can be regarded as, so to speak, “psychological health inequality from a positive psychological perspective”.

There is considerable evidence of the inverse relationship between poverty and mental health in high-income countries; the inverse relationship has been empirically demonstrated in low- and middle-income countries like Indonesia relatively recently [70,71,72]. Moreover, academic arguments on social determinants of mental health appeared around 1998 ([73]), and research has recently increased ([74,75,76,77]), partly impacted by the arguments on social determinants of physical health, for example, by Marmot ([78]).

Nevertheless, most research has been conducted independently from analyses of physical health inequality, such as independent variables. In addition, most research on mental health does not necessarily connect closely with psychological studies per se, particularly positive psychology. In fact, there is almost no literature entitled ‘social determinants of psychological health,’ although the word mental is frequently interchangeable with psychological.

In contrast, this study investigates psychological health inequality from the perspective of positive psychology in a comparative method to physical health inequality. It would be effective to analyze this psychological issue parallel to the research on physical health inequality because psychological health is somewhat related to physical health. 

Although there has recently been a considerable amount of literature on political philosophy and health inequality ([79,80,81]), now the classical *Is Inequality Bad for Our Health?* [38] still presents the central points of discussions, as a seminal article and a review (chapters 4 and 8 in [51], [82])on this theme substantially address the arguments of its authors. 

The title of *Is Inequality Bad for Our Health?* [38] implies the desirability to reduce social inequality to improve physical WB. However, questions have appeared regarding the propositions of the book. Although most researchers agree with socio-economic determinants of health, some doubt whether the factor is worthy of the most urgent attention.

First, although socio-economic factors influence health, it is unclear which aspect and to what extent it is within factors such as wealth, education, occupation, or other factors.

Secondly, though inequality seems to be a direct cause of poor health, the real cause may be poverty (Angell, pp. 44, 46, [83].)

Thirdly, the fair allocation of medical care and access to health care is more important than tackling social inequality as a policy option (Marmor, pp. 57–58, [84]; Emanuel, p. 66, [85]).

Fourthly, there are philosophical discussions about whether the prescription based on Rawlsian justice is appropriate. According to Rawlsian theorists, the difference principle (in the second principle) requires that a just society restricts inequalities in income and wealth to those that benefit the least advantaged (Daniels, Kennedy, and Kawachi, p. 19, [38]; Daniels, Kennedy and Kawachi, pp. 3–33, [55]). On the other hand, justice does not command the reduction of health inequality after we were to achieve a just society, namely, a just distribution of the resources for the least well-off (p. 23, [38], Daniels, Kennedy, and Kawachi [55]). 

Applying Rawlsian political philosophy in Daniel’s way has invited theoretical criticisms and alternative constructions by a few theorists [86,87,88]. For example, Shlomi Segall and others criticized Daniel’s argument on the relationship between principles of fair opportunity and difference [89,90]. This debate confirms the significance of Daniel’s propositions, but these theories, somehow based on Rawlsian philosophy, ‘are silent on the justice of residual health inequalities’ (Section 6.3 Derivative Approach in [82])after the realization of Rawlsian justice. 

So then, whether it is sufficient for us to focus on the worst-off individuals arises. According to Amartya Sen in the foreword of this book, ‘Concentrating merely on the worst-off individuals… give us, therefore, a less sensitive measure of inequality than we need for relating socioeconomic inequality as health inequality’ (p. xv, [38]). This issue indicates whether the Rawlsian conception of justice is sufficient for dealing with psychological disparity. 

The health inequality issue is so significant that it has been gradually recognized in political philosophy in general: Norman Daniels contributed the last chapter, ‘Individual and Social Responsibility for Health,’ to *Responsibility and Distributive Justice* [26]. Moreover, various issues of public health and ethics around health inequality have been explored in a few books [91], represented by Sudhir Anand, Fabienne Peter, and Amartya Sen, eds., *Public Health, Ethics, and Equity* (2004) [92], including chapters by the authors mentioned above, including Sen, Marmot, and Daniels/Kennedy/Kawachi. 

Nevertheless, the central issues enumerated in *Is Inequality Bad for Our Health?* have not been entirely resolved yet, and it would be valuable to analyze these with new data. In addition, Japan is often mentioned as one of the egalitarian countries with comparatively better situations concerning health and the concomitant social problems in the literature; it is worth examining its inequality problem.

Moreover, exploring the political dimension in the inquiry of social determinants of health is rare. In this respect, it is noteworthy that Daniel E. Dawes called attention to this aspect in his *The Political Determinants of Health* [93] two years ago (2020). Nonetheless, his book offers a theoretical perspective but does not contain his own empirical analyses. Therefore, this study, with empirical analyses, can contribute to the political determinants of psychological health in response to his proposal.

In addition, although he included political aspects such as government, voting, and policy from the perspective of equity/inequity, he did not primarily focus on justice and fairness in independent variables. Accordingly, as most research on the social determinant of health has not dealt with the ethical and political aspects of fairness/justice, introducing these factors would contribute to our understanding of health disparity.

## 3. Materials and Methods

### 3.1. Population, Questions, and Collection of Data

Three online surveys were designed to comprehensively study the relationship between individuals’ WB and the natural or social conditions surrounding those individuals: May 2020 with a sample size of 5000; March 2021 with a sample size of 6885; and October 2021 with a sample size of 2472(Appendix A.1). The responses were treated anonymously and tabulated (It was made clear that the survey results would only be used for related research, and neither the respondents’ personal information nor the content of individual responses would be used for any other purpose or leaked to any third party.). The statistical analyses focus on various WB and their relation to psychological health and their changes due to COVID-19.

These surveys were designed to investigate well-being, society, and politics in Japan comprehensively. Accordingly, the number of questions in Surveys 1 through 3 were 383, 401, and 174, respectively. Therefore, it contains many questions for identifying the factors that promote WB. However, the questions analyzed in this study are limited part of all.

The principal indicators used to measure the degree of WB concerning this paper were (Appendix A.2):

1 SWLS (5 questions)

2. PERMA profiler (23 questions)

3. I COPPE (19 questions)

4. Physical/Mental and Feeling Change under COVID-19

In the items above, SWLS in the list denotes the Satisfaction With Life Scale, developed by Ed-Diener [94], which has been the most popular index of subjective WB. This indicator is the life satisfaction component of subjective WB. 

PERMA, proposed by Seligman [4], refers to the following five components of WB: Positive emotion (P), Engagement (E), Relationship (R), Meaning (M), and Accomplishment (A). The PERMA profiler developed by J. Butler and M. Kern also includes health (H) and negative emotion (N) [95].

I COPPE in the above list was developed by I. Prilleltensky and colleagues [96] to assess the muli-dimensional WB in various domains in life (as this acronym indicates): Overall, Interpersonal, Community, Occupational, Physical, Psychological, and Economic WB. Original I COPPE asks about past, present, and future, but our surveys are limited to the last two because of the practical limit concerning the number of questions. Moreover, questions about the future ask “a year from now” in the original I COPPE; “five years from now” in our survey. This modification (from one year to five years) is to ensure that respondents consider their situation well after the COVID-19 problem.

The original scale of answers of SWLS is 7 points: PERMA profiler and I COPPE are 11 points (0 to 10). Nevertheless, the respondents were asked to choose one number for each question from 1 (not agree at all) to 10 (agree very much), with a few exceptional questions mentioned below. The financial reason required the unification of the format because of the cost estimate of the internet company. In addition, as the company’s format does not allow 0 as an option, it is not attractive to use 11 points scale (1 to 11): as the survey indicates only numbers except the poles (1 and 10 in 10 points scale), it is not easy to choose the neutral point intuitively in the case of 11 points scale.

In principle, the odd-number scale has advantages and disadvantages: while it lessens the psychological burden of respondents to choose other than a neutral point, it makes analyses difficult because the neutral answer allows various interpretations. Although the modification above has disadvantages for international comparison, the advantage of the even-number-point scale is especially great in Japanese surveys because Japanese people tend to choose the midpoint (for example, five on a scale from 1 to 10): “Neither agree nor disagree”. As this cultural tendency makes analyses difficult, these surveys choose an even-number-point scale as a unified format.

Nevertheless, original simple questions measuring mental change use the odd-number scale because it was supposed that many people feel that there has been no change: survey 1 asked about the mental change caused by COVID-19 (5 scales from 1 ‘have become very good’ to 5 ‘have become very bad’).

In addition, Survey 1 and Survey 2 asked about the existence of change of feeling or mood (yes or no) for a light feeling, dark feeling, anxiety, and feeling depressed (abbreviated as depression in the following) by COVID-19.

Questions concerning this paper are related to biological, natural, cultural, and socio-economic factors of psychological health. As biological factors such as healthy foods and exercise are regarded as indispensable, this paper analyzed the factors by the first two surveys (after Section 4.1) because Survey 3 lacks these factors in questions.

Survey 1, conducted in June 2020, collected responses from 5000 people living in Japan’s 47 prefectures. The breakdown of the respondents was 50% (2500) male and 50% (2500) female. Survey 2, conducted in June 2021, also targeted residents of Japan’s 47 prefectures as in Survey 1 and received responses from 6885 respondents. Of these, 64.3% (4427) were male, and the age range varied from teens to over 70s. Survey 3, conducted from 26 to 28 October 2021, targeted the same 47 prefecture residents of Japan as Surveys 1 and 2, and responses were collected from 2658 respondents. The male/female ratio was 66.2% (1759)/33.8% (899).

An Internet research company conducted the surveys: they motivated respondents by offering incentives (some points as rewards for purchase). However, as the original data collected online contained insincere responses, these were removed by a statistical standard. After the data cleaning, the number of respondents for Surveys 1, 2, and 3 was 4698 (the male/female ratio was 48.6% (2283)/51.4% (2415)), 6855 (the male/female ratio was 64.2% (4404)/35.8% (2451)), and 2472 (the male/female ratio was 65.8% (1626)/34.2% (846)), respectively (see Appendix B for details).

### 3.2. Data Analysis Method

WB is measured in this paper by SWLS, PERMA, and I COPPE. Moreover, PERMA and I COPPE include terms concerning psychological health. So, psychological health can be measured by general WB in the PERMA indicator and psychological WB in the I COPPE indicator. Then, this paper’s index of Psychological Health is constituted by the mean of general WB (in PERMA) and psychological WB (in I COPPE). Psychological Health will be abridged as PSH in the following.

First, some descriptive statistics were calculated for each Survey. In particular, the change concerning WB under COVID-19 was analyzed in Section 4.1.

Secondly, the relation between objective personal economic situations in income/assets and psychological health was examined in Section 4.2 in parallel to the health gap mentioned above in Section 2.2.

Thirdly, factors influencing psychological health were examined along the physical health inequity framework described above. Based on the results of previous studies on physical health, this research analyzed biological, natural, cultural, and socio-economic factors. Correlation calculations and multiple linear regression analyses (stepwise method, use of probability of F: entry 0.05, removal 0.10) were applied to estimate the impact of each factor in Section 5.1 and Section 5.2.

Fourthly, factors concerning mental changes or feeling changes under COVID-19 were analyzed. As a result, fairness and justice were focused on as social determinants of psychological health disparity, and their impacts on the level and the change of psychological health were investigated by multiple regression analyses and logistic regression analyses (backward elimination, likelihood ratio (stepwise method), use of probability of F: entry 0.05, removal 0.10) in Section 6.1, Section 6.2 and Section 6.3.

Statistical analyses were conducted using the statistical package SPSS (version 28).

## 4. Results 1: Psychological Health Inequalities concerning Objective Personal Economic Situations

### 4.1. Decline and Polarization in WB during the COVID-19

**Figure 1:**
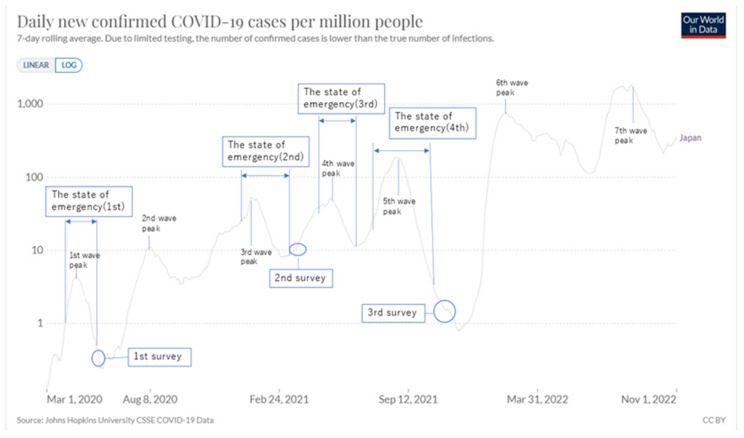
COVID-19 Phases in Japan. Note: The number of infections is indicated logarithmically. Source: Japanese situations are inserted into Our World in Date data. (https://ourworldindata.org/coronavirus#coronavirus-country-profiles, accessed on 19 November 2022).

Figure 1 (and Appendix C) indicates the phases and main critical situations concerning COVID-19 in Japan to offer the background of this study. The vertical axis of Figure 1 shows the daily new confirmed COVID-19 cases per million displayed in the log. There are five cycles during the period of three surveys.

The Japanese government issued mainly non-binding orders during this period: ‘declaration of a state of emergency,’ the stronger measure, asking people in the applied prefectures to stay at home, to cooperate with infection prevention measures, imposed restrictions on the use of facilities; ‘priority measures to prevent the spread of disease’ requesting restaurants to shorten their working hours until 8 p.m. and visitors to accept infection prevention measures, such as wearing masks.

In each cycle, these two measures began in some prefectures, sometimes extended to other prefectures, and ended in some prefectures, extending to all prefectures concerned. ‘Declaration of a state of emergency’ was extended to the whole country only in the first wave. Nevertheless, the fifth cycle was the most serious during this period, for example, in the number of new infections.

Survey 1 was conducted immediately after the first ‘state of emergency’ (7 April 2020~25 May 2020: in some prefectures, the same in the following) in the first wave. Survey 2 was conducted after the second ‘state of emergency (7 January 2021~21 March 2021) in the second wave. Survey 3 was conducted in the fifth wave after the fourth state of emergency (12 July 2021~30 September 2021).

**Figure 2:**
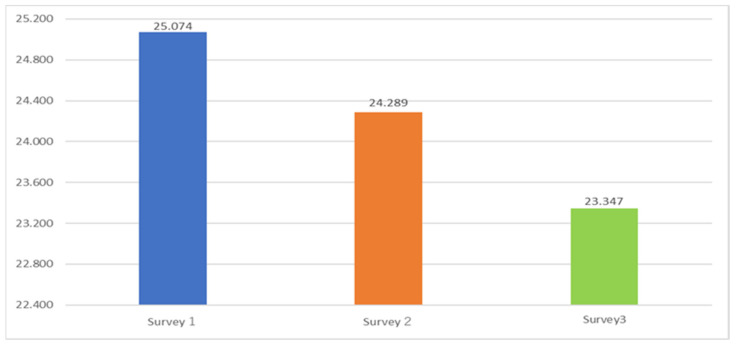
Comparison of WB in the Three Surveys (SWLS Index).

**Figure 3:**
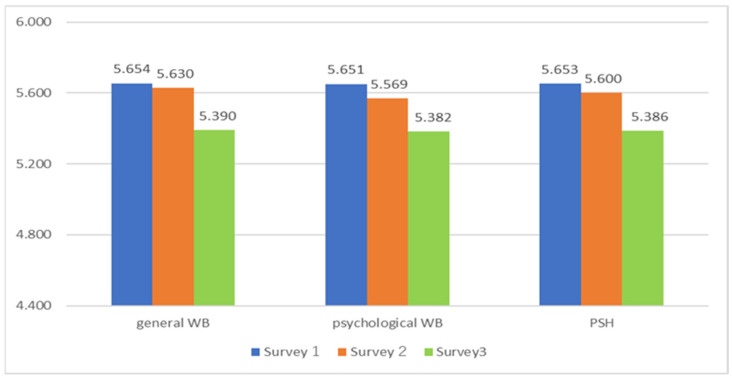
Comparison of Psychological Health Indices in the Three Surveys (general WB/psychological WB/PSH).

From Survey 1 to Survey 3, a continuous downward trend was detected in all of the measures examined, including SWLS, PERMA, and I COPPE. Figure 2 indicates SWLS, and Figure 3 indicates general WB in the PERMA profile, psychological health in I COPPE, and PSH. The differences between Survey 2 and 3 are larger than those between Survey 1 and 2: This fact may reflect the seriousness of the fourth and fifth waves before Survey 3.

This result is in congruence with academic surveys in the world [97,98,99] and Japan [100,101,102]. For example, according to official reports, WHO estimated a 27.6% increase in cases of major depressive disorder and a 25.6% increase in cases of anxiety disorders worldwide in 2020; Surveys by the Japanese Ministry of Health, Labour, and Welfare indicate that 40~50% of the population felt some anxiety during this period.

In terms of changes in mental health in the wake of the COVID-19 disaster, many respondents reported that their mental health had worsened, but a certain percentage of respondents’ mental health had improved. This observation indicates a trend toward polarization of WB over the survey period.

The results above indicate that analyses using PSH lead to mainly the same result as those using general WB and psychological WB. Accordingly, the following analyses utilize PSH.

### 4.2. Income and Psychological Health

**Figure 4:**
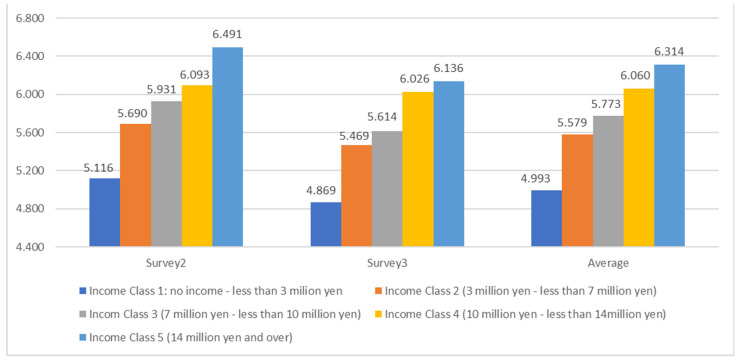
Comparison of Average Values of Psychological Heath by Household Income (Survey 2, 3). Note: Survey 2: Income Class 1: N = 1363, Income Class 2: N = 2731, Income Class 3: N = 1075, Income Class 4: N = 399, Income Class 5: N = 194, The responses “No idea” in item 22 have been removed. Survey 3: Income Class 1: N = 484, Income Class 2: N = 1015, Income Class 3: N = 385, Income Class 4: N = 164, Income Class 5: N = 101, The responses “No idea” in item 22 have been removed.

Survey 2 and Survey 3 asked about the annual income of an individual and his/her household. Figure 4 compares the two surveys and their mean about mean values of PSP, divided into five classes according to their degree of household income. It is clearly seen that the higher the annual income, the better the psychological health. The analysis of individual income also proves this tendency. As Survey 2 and 3 indicates the same tendency, it is reasonable to show it by their mean.

These figures indicate that psychological health inequality measured by WB indicators (as subjective measures) has a close association with economic inequality (as objective measures) in Japan; this finding is in line with existing studies on physical health outside of Japan [33,34,35,36,42,43]. Although the health measured here is the person’s subjective perception of psychological health (self-rated subjective health), this has been proven to be practical as a health indicator [103]. Thus, psychological health inequalities are related to economic factors.

Moreover, this result confirms that psychological health inequality is related not only to poverty but also to the whole range of economic inequality.

## 5. Results 2: Factors of Psychological Health Inequality

### 5.1. Correlations with Psychological Health Inequalities

As Survey 1 and Survey 2 have survey items on exercise and foods, we analyzed the factors enumerated in the discussion of physical health inequalities in the last sections: ascriptive factors, biological factors, natural and cultural factors, and social factors concerning economy, societal community, and politics. Therefore, this paper will call the factors enumerated below ‘basic factors’ in contrast to additional factors in the later sections.

Table 1 indicates correlations between PSH and the basic factors in the two surveys and the means of correlations between the two surveys.

1. ascriptive factors: sex, age, occupation, marriage

The correlations with attributes of sex and age in both surveys are less than 0.15 or insignificant. Only age has a weak association with PSH in the lower 0.1 range only in survey 2.

The two surveys’ correlations between marital status and PSH are lower than 0.25. In addition, the correlations concerning occupation (or no occupation) are below 0.25.

Accordingly, the correlations concerning ascriptive factors are low overall.

2. biological factors: exercise, foods, medical environment

The correlations in Survey 1 between “exercise (adequate exercise habits)” and PSH are in the higher 0.3 range, and the correlations concerning foods (healthy foods life or eating habits) are in the 0.5 range. In Survey 2, “exercise and foods” are treated together in one item and are 0.7 range. So then, the two surveys’ average correlations are below 0.6.

The two surveys’ correlations concerning the “medical environment” are in the 0.4 or 0.5 range.

Then, the biological factors have moderate but substantial associations with psychological health.

3. Natural and cultural factors: natural environment, educational environment

The two surveys’ correlations concerning the “natural environment” range in the 0.4–0.5 range. The two surveys’ correlations concerning the “educational environment (around oneself and children near them) are in the 0.5–0.6 range.

Previous studies on physical health have demonstrated that education and the natural environment relate to health inequality, and this study confirms the substantial relation concerning psychological health inequality.

4. Economic factors: income, assets, employment stability

The two survey correlations concerning “income”, “assets”, and “employment stability” are in the 0.4–0.5 range.

Accordingly, the relations regarding the economic factors are moderate but substantial, as expected. This result confirms the analysis in Section 4.2 using the objective income.

5. Societal community factors: stratification satisfaction, general trust, disparity recognition, disparity elimination

The two survey’s correlations with stratification satisfaction (satisfaction with social status and stratification, abridged as stratification in the following) are in the 0.6 range. The correlations with general trust (trust in people in general) are in the 0.5–0.6 range.

These correlations concerning societal-community factors are moderate, and those regarding stratification satisfaction are generally higher.

Further examining the relationship with disparity, the two survey’s average correlations with disparity recognition (in society) are in the 0.1–0.2 range. On the other hand, the total correlation with recognition of eliminating disparity (eliminating disparity and achieving an equal society through social welfare, redistribution through taxes, and so forth) ranges in the 0.3–0.4 range.

Accordingly, the correlations regarding the subjective disparity recognition or disparity elimination are significant but small; they are smaller than the two societal community factors above.

6. Political factors: fairness/justice, anti-corruptive fairness, human rights, and civil efficacy

The two survey’s correlations with fairness/justice (in Japanese politics in terms of decision-making, the disparity between the rich and the poor, and so forth) and anti-corruptive fairness (the country’s government is fair and not corrupt) are in the 0.3–0.4 range. The correlations are small or moderate but substantial.

The correlations concerning human rights are in the 0.4–0.5 range. As rights are the central conception of justice in contemporary mainstream political philosophy (liberalism and libertarianism), this item is also related to justice. The correlations regarding civil efficacy (possibility or wish to change the society and politics towards desirable directions by one’s own engagement) are in the 0.4–0.5 range: this factor is related to citizenship.

Accordingly, the correlations concerning human rights and civil efficacy are moderate, and these political factors, including fairness and justice, are small or moderate yet substantial.

**Figure 5:**
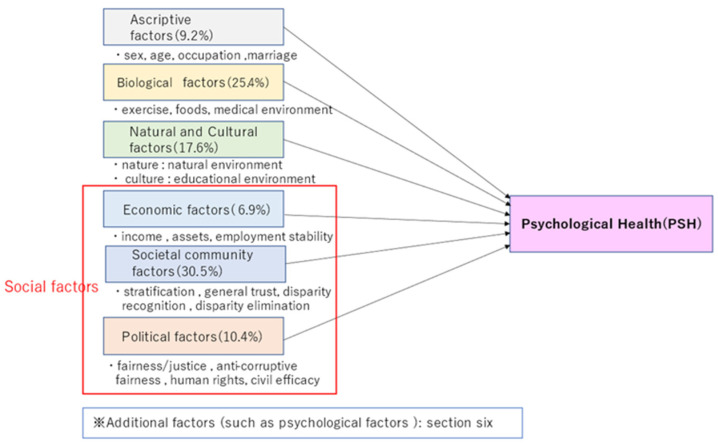
Psychological Health Inequality: Multiple Regression Analysis. Notes: This figure is a conceptual framework of the multi-regression analysis. This indicates all factors within each category, apart from the result of Psychological Health.

Table 1 indicates the rankings of the factors concerning PSH. In addition, Table 2 shows the average and rankings of the correlations concerning the categories of factors. Colors in the following tables and Figure 5 correspond to categories.

Thus, as was pointed out in the discussion of health inequalities, social factors are pretty significant in the inequalities. According to Table 2, biological factors are prominent (second or third) factors in parallel to the case of physical health examined in previous studies.

Next, natural and cultural factors (natural and educational environment) are highest in the two surveys.

Thirdly, although the societal community category is the fifth or fourth in Table 2, concerning each factor, stratification is the highest or the fourth in Table 1: its average is the highest, even exceeding biological factors and natural/cultural factors.

Fourthly, the other social factors have moderate correlations next to the three categories. In fact, economic factors (income, assets, and employment stability) and political factors (human rights, civil efficacy) are comparable in magnitude to some biological factors (Table 1).

The category of political factors has the 0.4 range (Table 2). The correlations with both disparity recognition/elimination and fairness/justice are less than the biological factors (Table 1). Nevertheless, as human rights are essentially equal to legal justice, some factors in fairness and justice play a substantial role in predicting psychological health.

### 5.2. Multiple Regression Analyses on Psychological Health

Then, multiple regression analyses on these factors have been conducted in order to analyze the relative importance of these basic factors in predicting psychological health. Appendix D shows the results, and Table 3 summarizes them. The latter indicates the ranking and relative importance (*β*) of factors concerning PSH; The colors of each cell indicate categories.

In the following, R2s are moderate; factors will be enumerated mainly from the highest positive factors in the following.

Those with higher β are stratification, foods, general trust, natural environment, civil efficiency, natural environment, human rights (over 0.1), educational environment, medical environment (over 0.07) in Survey 1; exercise/foods (about 0.3), educational environment, general trust, stratification, medical environment (over 0.1), natural environment (over 0.07) in Survey 2.

Therefore, these factors can be classified as the first group of factors: the dividing line in Table 3 roughly indicates this group as above the line. Accordingly, biological, societal community, natural/cultural factors, and political factors are the four essential factors in psychological health.

To a lesser degree than these, there are assets or income, marital status, occupation, sex (over 0.02 in both surveys), employment stability, and disparity recognition (over 0.03 in either survey) (Appendix D). So then, these factors can be classified as the second group of factors, which are below the dividing line in Table 3. Accordingly, economic factors, some ascriptive factors (marital status, sex), and some social factors (disparity recognition) are related to the psychological health next to the first group.

Therefore, biological factors or stratification are the most effective, and the other natural/cultural and social (societal community, economic, and political) factors play a substantive role in psychological health.

Table 4 summarizes these results as the relative ranking of categories. This ranking was the estimation by the two survey percentages of the standardized partial regression coefficients (*β*) of the factors within categories. As the number of factors varies between Survey 1 and 2, their values and the mean of the two surveys should be regarded as only a rough approximation of the relative importance of categories. Table 4 includes the average of two surveys about the sum concerning all basic variables within each category, while Appendix E.1 is about the sum concerning the top two variables: these tables are shown just for information on trial calculations.

Nevertheless, as the rankings of the two surveys are similar, this result is robust. Moreover, the rankings concerning the average of the two surveys are close to those of each survey. Accordingly, the calculation of the average proves to be effective despite the time difference. Therefore, the average will be sometimes indicated hereafter instead of writing the results of the two surveys for convenience.

First, the societal community factor is the highest (Survey 1 and average). This remarkable result indicates the importance of socio-community factors such as stratification.

In addition, it is noteworthy that the economic factor is sixth (Survey 1 and average), less than the other categories. In other words, natural and cultural, societal community, and political factors are more associated with psychological health than economic factors.

Moreover, political factors, including justice (human rights) and citizenship (civil efficacy), are the fourth (Survey 1 and average).

On the other hand, fairness/justice does not appear as a positive health factor: in particular, anti-corruptive fairness mainly has a negative partial regression coefficient in psychological health (Appendix D). This negative association is contrary to the original theoretical supposition before calculation. However, this may be because people who realistically acknowledge Japanese society and politics tend to recognize corruptive unfairness but can hold better WB because their understanding is sober or reasonable.

Thus, psychological health is closely connected with biological, natural, cultural, and social factors: Figure 5 exemplifies the association by illustrating the result of multiple regression analysis concerning the average of the two surveys. As social factors consist of economic, societal community, and political factors, these can be called socio-economic and political factors or simply socio-economic-political factors hereafter.

## 6. Results 3: Psychological Dynamics under COVID-19

### 6.1. Factors Concerning Mental Changes under COVID-19: Appearance of Disparity Recognition and Fairness/Justice

Survey 1 includes items on mental changes under COVID-19, which enable us to analyze the relationship between psychological changes and the factors above.

First, those with correlation coefficients of −0.1 or lower with regard to mental changes (high value signifies the bad direction) are exercise, foods, educational environment, income, assets, employment stability, stratification, general trust, fairness/justice, anti-corruptive fairness, and civil efficacy. Perhaps because of correlations about change, the values (in the lower −0.1 range) are small (Table 1), with about the same for biological factors and social factors such as economic, societal-community (stratification, general trust), and political factors: in contrast, natural and cultural factors are relatively small. In particular, it is worth noting that anti-corruptive fairness and fairness/justice, which were smaller in value than the major factors in the above analysis, are about the same here.

Regarding psychological change, categories from the highest are economic, political, natural/cultural, biological, societal community, and ascriptive (Table 2). As economic factors are the highest and political factors are the third or the second, the relative significance of these social factors rises in this analysis of the dynamic change compared with the analyses above.

Thirdly, the same multiple regression analysis as above (Table 3) shows that for mental change (adjusted R-square of 0.040), items from the largest absolute value of β to the smallest are stratification, exercise, anti-corruptive fairness, disparity recognition (opposite sign to the other items), sex (male), employment stability, and young age. While only disparity recognition worsens the situation, the other factors facilitate the desired change or suppress the undesirable change in the physical/mental change.

In these cases, the R-squared and overall values are small (below 0.1, except for age). However, disparity recognition, fairness/justice, and anti-corruptive fairness emerge as significant factors in addition to the factors appearing in the multi-regression analysis in Section 5.2. Moreover, the absolute value of anti-corruptive fairness and disparity recognition are the third and the fourth concerning mental change. Accordingly, political factors are the fourth (Survey 1, average) in the ranking of categories; the societal community is the first, while economic factors are the fifth (Table 4).

Looking back, fairness/justice and anti-corruptive fairness are substantial and comparable factors in magnitude to the other vital factors, but disparity recognition is a minor factor in correlation analyses in Section 5.1. In addition, even fairness/justice and anti-corruptive fairness are not some of the most significant factors in the multiple regression analysis in Section 5.2. Therefore, it can be inferred as follows: in a crisis such as COVID-19, disparity recognition, fairness, and justice play a more significant role than their general contribution to psychological health in usual times in order to deter the decline of the WB.

### 6.2. Factors Concerning Feeling Changes under COVID-19: Importance of Disparity Recognition, Fairness, and Justice

Survey 1 and Survey 2 asked about the change of feeling or mood for light feelings, dark feelings, anxiety, and being depressed under COVID-19 (Appendix A.2). So then, these items enable us to analyze the relationship between psychological changes and the above factors. Nevertheless, as answers to these items are existent (yes) or non-existent (no), logistic regression analysis is required. Table 5 indicates the significant ascriptive factors and the other factors in the ranking (based on Appendix F and Appendix G). Table 6 and Appendix E.2 indicate the ranking of categories concerning this analysis by the method identical to Table 4 and Appendix E.1.

First, in the correlational analysis (Table 1), the two survey’s average correlations between change for light feeling and the other three feelings for the undesirable direction are weak (the −0.1 range). On the other hand, those among the latter three items are moderate (the 0.3 range). Those between change for a light direction and mental change in Survey1 are small (around −0.1), while those concerning the other changes for undesirable direction are a bit larger (the 0.1–0.2 range).

Secondly, there are no items with the two survey’s average correlation coefficients higher than 0.1 about a light feeling; items with correlation coefficients lower than −0.1 about feelings for undesirable directions (more than one item) are exercise/foods, medical environment, educational environment, income, assets, employment stability, stratification, general trust, disparity elimination, fairness/justice, anti-corrupted fairness, human rights, and civil efficacy.

Notably, disparity elimination instead of disparity recognition in the last section appears in this list; fairness/justice and anti-corruptive fairness concerning all items for undesirable directions is lower than −0.1.

Thirdly, logistic regression analyses (Table 5: Survey 1 and Survey 2-1) indicate the following facts, although N2 (of Cox and Snell, Nagelkerke) is small. As the values concerning light feelings are minimal, analyses of the negative direction changes are cardinal in the following. In addition, ascriptive factors of sex, occupation, and age should be estimated independently because the forms of answers to these are different from the others. In particular, females’ feelings tend to turn in negative directions.

Then, regarding the increase of light feeling, items concerning the correlations above 0.1 are, from the highest, educational environment and human rights in Survey 1; those are civil efficiency and stratification in survey 2.

Next, regarding the increase in undesirable feelings, items concerning the correlations above 0.1/below −0.1 more than one feeling among the three (dark feeling, anxiety, and depression) in two surveys are disparity recognition and anti-corruptive fairness.

Therefore, political factors play vital roles in these analyses. For example, the item of human rights and civil efficiency are two of the four factors above concerning the change for light direction: disparity recognition and anti-corruptive fairness are all factors concerning the undesirable directions when the ascriptive factors are excluded. In fact, political factors in ranking categories of these feelings are the first or the second in Table 6.

Moreover, the number of appearances of disparity recognition, fairness/justice, and anti-corruptive fairness as variables in results concerning undesirable directions is 6, 1, and 4 times, respectively, within six analyses in Table 5 (Survey 1 and 2-1: Appendix F). Thus, this analysis’s political factors regarding fairness and justice are vital.

### 6.3. Pivotal Factors of Fair/Just Society and Distributive Justice in the COVID-19 Crisis

As fairness and justice are essential factors, Survey 2 increased related questions. Therefore, although the analyses above are restricted to items common in both surveys, the final analysis introduces additional new items (Appendix A.1): fair society (‘Considering all, I think society now is fair’), just society (‘Considering all, I think society now is just’), fair/just society (mean of a fair society, unfair society(−), just society, and unjust society(−)), and distributive justice (mean of three items concerning the realization of distributive justice). Then, independent variables are all basic factors, including ascriptive factors, and these added new factors.

First, Table 1 also indicates their correlations. Correlation coefficients between fair society/just society and psychological health are in the 0.4 range, more than fairness/justice and anti-corruptive justice used in the analyses above (Table 1). On the other hand, correlation coefficients between fair society/just society and the changes in feelings are approximately 0.05 (change for light feeling) and the −0.1 range (changes for the undesirable directions): these are a little more than those of fairness/justice(Table 1).

Secondly, multiple regression analyses of the relationship between various factors and psychological health lead to similar results to those in Section 5.2 about Survey 2.

Thirdly, logistic regression analyses highlight the vital role of political factors such as fairness/justice in the change of feelings more clearly than the results in Section 6.2, though R2 (of Cox and Snell, Nagelkerke) of these analyses are still small (Table 5 Survey 2-2).

Like the last section, the ascriptive factors of sex, age, and occupation should be estimated independently: females’ feelings in all four questions tend to change in a less favorable and more unfavorable direction than males.

Among the other factors, civil efficiency is the first factor in the magnitude of absolute value in β concerning light feeling. Disparity recognition and anti-corruptive fairness are the first and third factors concerning the dark feeling. Anti-corruptive fairness, distributive justice, disparity recognition, and a fair/just society are anxiety’s first, third, fifth, and seventh factors. Finally, distributive justice, fair/just society, human rights, and disparity recognition are the second, third, fifth, and seventh factors concerning depression.

In addition, It would be reasonable to assume that psychological factors such as optimism, will to contribute to people and society, and hedonic or eudaimonic orientation in WB (measured by Veronika Huta’s Hedonic and Eudaimonic Motives for Activities: Revised HEMA-R [104], Appendix A.3) also influence the psychological health; similarly, these and the level of psychological health influence the mental change. Then, adding these factors (Appendix A.1) into the independent variables above in the multi-regression and logistic regression analysis leads to an increase in N2, as Appendix H and Appendix I show.

As a result of these analyses, the appeared variables are fair/just society in Appendix H; disparity recognition (four cells), anti-corruptive fairness (five cells), human rights (two cells), civil efficiency (two cells), distributive justice (two cells), and fair/just society (one cell) in Appendix I (Cells are counted only when the sign(+ or −) of correlations is the presumed direction.). In sum, this result is principally the same as immediately before (Table 5 Survey 2-2) except for the added factors in the undesirable directions.

Consequently, it is imposing that the factors regarding fairness and justice, particularly distributive justice, are some of the highest factors among the biological, natural, cultural, or social factors. Therefore, these results enable us to recognize the pivotal function of political factors in the dynamics of feeling changes in the COVID-19 crisis, particularly that of fairness and justice in mitigating the damage of feelings.

## 7. Discussions on Multi-Dimensional Dynamics of Health Disparities

### 7.1. Multi-Dimensional Inequalities/Disparities and Policy Implications

Although Japan is often considered one of the better countries concerning health inequality, this study demonstrates its existence in Japan: the results are mainly in line with previous studies ([105,106,107]). Moreover, current neo-liberal or market-oriented economic policies have increased inequality in Japan [108]. The recent socio-economic change may enlarge the issue.

The results of the analyses of the three surveys are almost the same as the tables and figures indicate. As was mentioned in Section 4.1, Survey 2 was conducted one year after Survey 1, and there was a severe life of COVID-19 for people between the two surveys (the second wave). This similarity of the results of multi-regression or logistic regression analyses demonstrates the robustness of the results. Thus, the results of this study offer some implications concerning the health inequality issues mentioned in Section 2.3.

First, this investigation empirically has demonstrated the relative weights of factors classified into biological, natural/cultural, and social factors in psychological health inequality. While most former studies on health inequality concentrate on physical aspects, the new finding here is that similar factors are significant in psychological health. These factors influence psychological health inequalities, just as they do physical health. Therefore, the new findings concerning psychological health disparity can contribute to the discussions on physical/psychological health inequality.

Secondly, although poverty is undoubtedly one of the causes of health inequality, as Section 4.2 demonstrated, not only poverty but also objective economic gradient as a whole have clear correlations with psychological health. This finding is in tune with the results of previous studies that the socio-economic gradient accompanies a continuous and stepwise gradient in objective physical health outcomes, such as morbidity and mortality, across the whole social hierarchy (pp. 10–11, [41]).

Thirdly, while some health inequality arguments focus on economic inequality, this study points to the significance of the other dimensions concerning inequality: not only income and assets but also the other social factors are associated with psychological health inequality. In particular, the significance of societal community factors aligns with the psychosocial hypothesis in physical health inequality: stratification and general trust correspond to social status and social network or capital.

Moreover, according to the correlation analysis in Section 5.1, the category of the economic factor is either the second or third highest (Table 2): less than natural/cultural factors, almost as much as biological, and more than the societal community and political factors; in contrast, multi-regression analysis in Section 5.2 demonstrates that biological and societal community factors are the highest or the second highest and the other categories, including political factors, are more significant than the economic factor, which is the lowest or the second lowest (Table 4). This difference indicates that the other categories are even more essential than the economic category, at least in subjective perception. It follows that substantial parts that previous physical health inequality arguments ascribe to economic inequality are more closely related to natural, cultural, and socio-political factors than economic factors per se.

Therefore, not only economic but also natural, cultural, and socio-political gradients are related to health disparity. As far as political factors are concerned, this study illuminated that these are as significant as the other factors.

Fourthly, although the medical environment is one of the substantial factors (eighth or tenth in the ranking of Table 1), other essential factors are equal to or more than that. As the quantity of resources for improving the latter does not seem to be necessarily more than the former, it would be desirable to execute public policies both for the medical environment and other factors.

These empirical arguments on factors concerning psychological health inequality signify that the causes of their inequality are neither limited to the economic nor the medical gap. They are also associated with natural, cultural, and social inequalities.

Moreover, considerable parts of these may also be disparities defined above because they might be avoidable and ethically unjust/unfair. Although it is unclear from the onset whether and to what extent some specific inequalities should be reduced, this is at least a theme of ethical and philosophical sincere debates. Therefore, it would be appropriate to term these factors regarding psychological health inequality as ‘multi-dimensional psychological health disparity,’ which relates to biological, natural, cultural, and socio-economic-political dimensions.

### 7.2. Philosophical Implications: Multi-Dimensional, Multi-Layered, and Ethical Fairness/Justice against Psychological Health Disparity

The fourth philosophical issue mentioned in Section 2.3 relates to the second, third, and fourth points above.

As social factors influence psychological health disparity, improving health care based on the Rawlsian principle of fair equality of opportunity is insufficient. Therefore, when arguments on physical health apply to psychological health, Daniels’ early argument [53] is not enough, and the application of the difference principle is required, as most theorists, including Segall [89,90] as well as recent Daniels [54], recognize.

It would be more logical for such Rawlsian theorists to concentrate on improving the conditions of the poor because their second principle of justice requires people to focus on the least advantaged. However, as discussed above, the empirical analyses indicate it is desirable to alleviate the socio-economic-political inequality in the whole range beyond the concern for the weakest.

This requirement transcends that of Rawlsian justice, as was mentioned by Sen (Section 2.3). Accordingly, this paper proposes the following three philosophical arguments beyond the Rawlsian health inequality argument.

First, the Rawlsian difference principle (within the second principle of justice) usually treats economically poor people and less socially advantaged ones associated with economic problems. Nevertheless, as Sen suggested by his capability approach, some lack the basic capabilities concerning cultural and social spheres other than the economic ones. In fact, the multi-regression analyses of factors concerning the psychological disparity in Section 5.2 demonstrate that, although economic factors are undoubtedly important, the other natural, cultural, and social or political factors are equal to or even more than them in their association; moreover, these other factors are not simply the mediating factor between economic factors and the psychological disparity but relatively independent factors along with economic factors. Therefore, tackling the multi-dimensional disparity beyond the simple economic dimension would be indispensable.

Secondly, although Rawlsians may regard this first proposal as the extension of the difference principle, alleviating inequality in the various spectrum beyond the policies focusing on the least advanced is more desirable about the multi-dimensional disparity. This issue of equality not restricted to the poor can also be just that of fairness in a broad sense.

Although Rawlsians conceptualize their principles as ‘justice as fairness’ [29,109], fairness in its common usage frequently implies some level of equality across the whole stratifications and classes: against excessive disparity itself in a multi-layered society. Therefore, equalizing the whole hierarchy to some extent in multi-dimensional disparity would be a philosophically cardinal agenda to be challenged for solving the issue of psychological health disparity.

Thirdly, this study demonstrates the significance of societal community factors such as stratification and general trust, and this finding corresponds to the significance of social networks and social capital in the psychosocial hypothesis of health inequality. These communal or relational aspects are, in reality, more consistent with communitarianism than individualistic libertarianism and Rawlsian liberalism.

Fourthly, the fairness/justice issue classically includes the ethical dimension. As Rawlsians regard justice as almost equal to rights, they separate justice as fairness from ethical dimensions like a good life. Nevertheless, the results indicate that although anti-corruptive fairness is negatively associated with psychological health (Section 5.2), it often mitigates the mental change and psychological changes for undesirable directions (Section 6.1 and Section 6.2): in the highest ranking cases (Table 5), the first (anxiety in the second Survey) and the second (depression in the first Survey).

This fact implies that the ethical dimension of fairness involved in anti-corruptive fairness is vital in times of crisis. This role is as significant as economic factors, such as employment stability, and social factors, such as disparity recognition; it seems to be more than biological, natural, cultural, and societal community factors, which are prominent in usual times.

Therefore, the ethical factor of fairness plays a crucial role in the dynamic analysis in Section 6 rather than the static analysis in Section 5: anti-corruptive fairness has a small or moderate correlation with psychological health but a considerable coefficient in multi-regression analysis. The reason for its increase in the dynamic analysis may be that people who believe in anti-corruptive fairness in politics can maintain hope and, therefore, their psychological health.

As communitarian political philosophy evaluates communality and ethicality in arguments for justice, the third and fourth point increases their plausibility in opposition to Rawlsian liberalism [12]. In concomitant with this, it would be better to regard justice and fairness as ‘ethico-political factors.’

In sum, the multi-dimensional, multi-layered, and ethico-political conception of justice and fairness would be effective for exploring to resolve the psychological health disparity.

### 7.3. Dynamism in the COVID-19 Crisis: Critical Significance and Causality of Fairness and Justice

Furthermore, this study explores the dynamism in the psychological changes during the COVID-19 crisis. Although political factors can be discerned in predicting psychological health disparity, they are relatively inconspicuous among the other prominent factors: the fourth in Table 4. However, the analyses in Section 6.1, Section 6.2 and Section 6.3 demonstrate that political factors play a salient role in the mental change and change of feelings: the first or the second (undesirable feelings). Moreover, not only the ethical aspect discussed in the last section but also other items of fairness and justice, such as fairness/justice, distributive justice, and fair/just society, are significant in the dynamic analysis.

The questions on fairness and justice do not mention specific policies concerning COVID-19. Instead, most questions asked about general perception concerning these, and one (fairness/justice) referred to ‘decision-making and disparity between rich and poor’ (Appendix A.1). Nevertheless, as the surveys were conducted under COVID-19, the answers can be interpreted to reflect both people’s general impression of these and the government’s policy concerning COVID-19.

The finding above is itself noteworthy, but it also leads us to the following crucial reasoning. Since fairness and justice are associated with not only the level of psychological health but also its change, it can be presumed that this relationship is not only a correlation but also a mainly unidirectional causality. While the multiple regression or logistic regression analyses were based on this assumption, the analyses themselves do not settle the causality. Nevertheless, the following reasoning is plausible.

As long as the static analysis of the level concerning psychological health is concerned, causality between fairness/justice and psychological WB can be in both directions. In other words, there can be both causalities. On the one hand, objective or subjective recognition of fairness/justice increases WB in persons: fairness/justice → WB. On the other hand, high WB positively influences the perception of the level of fairness/justice (subjective fairness/justice) in society: WB → fairness/justice. In the latter direction, high WB tends to better subjective levels of various factors: in short, happy people tend to think the various surrounding environments are comfortable.

Among these two directions, the dynamic analysis shares the first: the objective fairness/justice in public policies for alleviating the damage caused by the pandemic or subjective perception of their existence may mitigate the negative psychological changes, namely: “fairness/justice → change in WB” This objective influence of fairness/justice is conceivable since mitigating the pandemic is the most critical policy issue.

In contrast, regarding the second direction, when negative psychological changes happen in persons, these subjective changes do not tend to cause the objective decrease of fairness/justice in society because the negative WB changes caused by the pandemic do not decrease objective fairness/justice. The subjective decrease of WB in persons could cause their perceptional decrease of subjective fairness/justice in society, but the extent may be small because most of them ascribe the subjective deterioration of WB to the pandemic rather than fairness/justice in society. Moreover, while the subjective decrease of WB caused by the pandemic tends to cause the subjective decrease of various factors around them, the decrease in fairness/justice may not be comparatively more significant. However, fairness/justice is the most striking factor in the logistic regression analyses. Therefore, the causation that the decrease in WB leads to the decrease of subjective fairness/justice” (WB → subjective fairness/justice)under COVID-19 would be unlikely or relatively minor.

Consequently, it can be reasonably conceived that objective or subjective fairness/justice weaken psychological aggravation during the crisis.

## 8. Limit of This Study

As for some limitations of this study, although only some income/assets questions are subjective and objective, the other survey items here are about subjective perceptions. Accordingly, like various previous studies of health inequality (p. 38, [41]), research on objective facts about psychological health and factors would be desirable: for example, the influence of various objective factors on objective health.

Secondly, as the base of this paper is online surveys through an internet survey company, this study contains methodological limitations. For example, the company gathered respondents by offering purchase points; this is not a randomized survey. Therefore, it would be desirable to test these results with randomized surveys in the future.

Thirdly, self-reporting questionnaires like these surveys can contain self-biases. Again, this problem is well-known in psychology, and most positive psychological studies are based on such questionnaires. As this survey method has advantages and disadvantages [110], it would be desirable to verify the results by other survey methods than self-reporting surveys.

Fourthly, this study analyses the data collected in all prefectures in Japan as a whole because this intends to investigate the general tendency and factors concerning WB mainly in a scale of the nation-state. Accordingly, this did not scrutinize the influence and differences of areas within Japan. However, as there are differences regarding residence (prefectures with or without big cities: Appendix B) between the three surveys, the robustness of the results seems to demonstrate that this factor does not affect the main results much. Nevertheless, since the influence of COVID-19 differed in various prefectures, as Appendix C indicates, analyzing this factor will be a task in the future.

Fifthly, the data in this paper was collected in Japan during the pandemic, and it is necessary to conduct research in other regions and situations. Accordingly, the results of this analysis, for example, the relative weight of various factors in their influence on psychological health, should not be universalized. Therefore, it would be helpful to compare the results of this study with various studies in other areas and dates. Moreover, the weights perhaps change according to areas, dates, and related conditions. This point is worth pursuing further.

Accordingly, the importance of social factors is to be scrutinized by surveys in other regions, considering cultural, social, or political differences. For example, the degree of social influence, including peer-to-peer comparison and screening among its citizens, appears relatively strong in Japan under COVID-19 [111]. From a comparative perspective, the feature of Japanese reactions to COVID-19 is weak “administrative control” and strong “social control”: The latter refers to control at the civil society level, or “social consciousness”, in contrast to direct intervention and control based on laws and ordinances, such as the Cabinet Order in the former. As Japan’s “declaration of a state of emergency” remains a non-binding declaration, this measure corresponds to weak “administrative control”. Since a person’s own WB is determined by his/her relationships with others, people in Japan with strong “social control” are more likely to increase their WB if their social relationships are good and vice versa. On the other hand, Japanese people may not react to government regulation as much as people in other countries with strong “administrative control“. These differences may influence the association of social and political factors with psychological health differences.

## 9. Summary and Implications: Protective Intervention on Multi-Dimensional Disparity for Fair/Just Society

This paper illuminated the multi-dimensional factors of the psychological health inequalities/disparities under COVID-19 by introducing positive and social aspects of WB into measurement: there are, of course, biological factors, but the importance of natural, cultural, and social elements was verified. This result is clearly similar to previous studies on physical health. Accordingly, the essential finding concerning psychological health in this study can be regarded as a common fact both in physical and psychological health.

**Figure 6:**
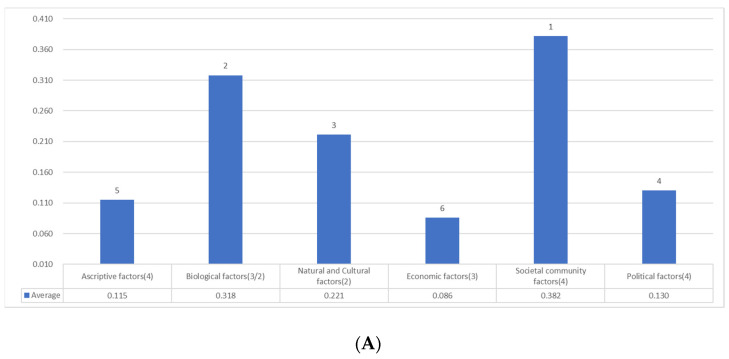

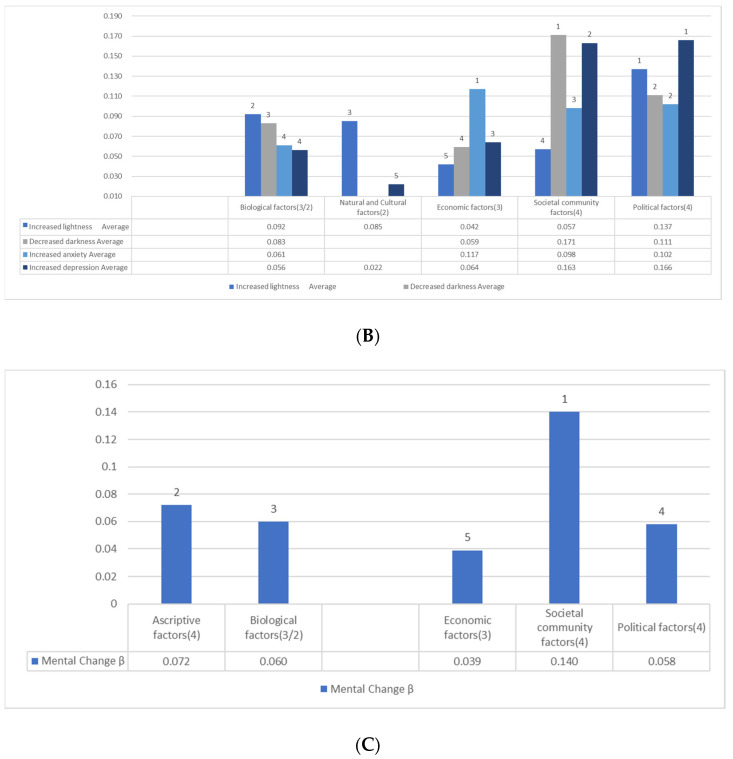
(**A**) Multiple Regression Analysis: Comparison of the Total Values (average) of Standardized Partial Regression Coefficients in Each Category. Notes: Figures in the graph denote each factor’s rank (from the highest). Figures in parentheses denote the number of variables in each category. As for derived (calculated) variables, refer to Table 4. (**B**) Logistic Regression Analysis: Comparison of the Total Values (average) of Partial Regression Coefficients in Each Category. Notes: Figures in the graph denote each factor’s rank (from the highest). Ascriptive factors are omitted since a simple comparison of the total values is not feasible due to different answer formats. As for calculated values, refer to Table 6. (**C**) Multiple Regression Analysis: Comparison of Standardized Partial Regression Coefficients Concerning Mental Change in Each Category. Notes: Figures in the graph denote **e**ach factor’s rank (from the highest). Natural and Cultural factors are not indicated since they do not appear as variables in the calculation result. Figures in parentheses denote the number of variables in each category. As for derived (calculated) variables, refer to Table 4.

In addition, Table 4 summarizes each factor’s relative importance, and the rough numbers concerning Psychological Health in units of 2.5% are 25%, 30%, 17.5%, 10%, and 7.5%; in short, the ratios are 10 (biological):12 (societal community):7 (natural and cultural):4(ascriptive, political):3 (economic). Moreover, Figure 6 illustrates the importance of categories both in static analysis and dynamic analyses: regarding the static analysis, Figure 6A is about PSH, while regarding dynamic analysis, Figure 6B is about the changes in feelings, and Figure 6C is about mental change. Figure 6A illustrates the ranking: ① societal community, ② biological, ③ natural and cultural, ④ ascriptive, ⑤ political, ⑥ economic. Figure 6B,C offers the impression. Excluding the ascriptive for comparison, Figure 6B ① political, ② societal community, ③ biological, ④ economic, ⑤ natural or cultural; Figure 6C ① societal community, ② biological, ③ ④ political and economic.

Therefore, while biological factors are the second or third in these analyses, societal community factors are the first or second. Political factors drastically rise in the dynamic analysis, as the first in Figure 6B, and even the third or the fourth in Figure 6C, while economic factors are below or near the political. Finally, natural and cultural factors are the third in the static analysis but disappear in the dynamic analyses.

Reflecting on these results, the cardinality of the societal community factor is congruent with the psychosocial thesis, and the significance of that and the political factor is consistent with the corresponding communitarian political philosophy.

It would be necessary to note that this calculation and ranking is merely an approximate estimation or impression, but the following remarkable conjecture is reasonable: while natural and cultural factors decrease in the dynamic analysis, social factors increase their significance in the dynamic analysis; political factors particularly increase their influence with stably important societal community factor.

Consequently, substantial parts of psychological health inequalities are associated with cultural or social structures in static and dynamic analyses. Therefore, these are also health disparities because they can be unjust or unfair and are avoidable: although the influence of some ascriptive factors, such as age, is unavoidable to some extent, collective human efforts can change most cultural or social conditions.

Now that the multi-dimensional factors are demonstrated to be more or less related to the disparities, it would be desirable to ameliorate these gaps in standard time. As discussed in Section 7.2, this paper proposes a multi-dimensional and multi-layered approach to psychological health disparity: this tries to improve biological, natural, cultural, and socio-economic-political situations along the whole strata. These factors are protective and facilitative for improving the disparity. The social factors consist of economic, societal-community, and political factors. Fairness and justice were highlighted in political factors, which are some of the substantial factors of psychological health disparity.

Nevertheless, things are different in times of crisis. It would be, of course, effective to improve all of the multi-dimensional factors to deal with the psychological crisis. However, under the COVID-19 pandemic, it is frequently difficult to do so because of various behavior restrictions. For example, regarding the biological, natural, and cultural factors, it would certainly be significant to recommend people exercise, diet, contact with nature, and cultural activities, but they are less easy than usual. Moreover, short-term changes in stratification satisfaction and general trust are difficult regarding societal community factors.

Economic interventions by politics are especially urgent when the pandemic prevents the usual economic activities. Moreover, beyond these measures, this study highlights the associated essential factor: fairness and justice. These are closely related to equality, and public policies for fairness and justice overlap with those for equality to some extent. However, these do not require complete equalization because some kind of inequality can be regarded as fair or just when the disparity reflects ethical deserts of peoples’ efforts to contribute to others and society.

Moreover, these ideas contain some ethical moments seen in anti-corruptive fairness. This element cannot be reduced to the economic dimension. People who regard the present society/politics as fair or just can have hope even in times of crisis. This psychology is perhaps a major reason why their perception of fairness and justice in society contributes to softening the drop in psychological WB. When people believe that their society is fair and just, they can maintain their psychological health and make efforts to overcome in front of unnormal difficulties. Therefore, the improvement of fairness and justice, at least in people’s perception and cognition, could be a new way of intervention to protect psychological health.

In fact, this study has empirically demonstrated that psychological health has been declining, especially with the prolonged COVID-19 problem; under this circumstance, there is a positive effect of fairness/justice on less deterioration of WB. This analysis thus demonstrates the protective importance of fairness/justice for psychological health. The realization of fairness/justice can be reasonably expected to increase people’s psychological health; in a crisis like COVID-19, it mitigates negative psychological changes. Therefore, it can be concluded that realizing a fair/just society generally increases people’s psychological health and suppresses its changes in the case of a global pandemic.

## 10. Conclusions

The World Health Organization (WHO) Charter states that health is a state of complete physical, mental and social WB and not merely the absence of disease or infirmity. This empirical analysis underpins the very Charter of the WHO. Furthermore, in an article introducing the Sustainable Development Goals (SDGs) (https://sdgs-support.or.jp/journal/goal03/, accessed on 19 November 2022), Goal 3 reads: “Health and Well-being. for All”, implying that there remain international as well as national disparities in health that need to be corrected. Therefore, an international comparative study of WB with a focus on social factors would be meritorious.

In this context, this article has empirically investigated the factors of psychological health inequality/disparity in the crisis of COVID-19 in Japan, and the main findings are as follows: there are multi-dimensional factors, including socio-economic-political factors. Not only economic factors but also ethico-political factors such as fairness and justice in the social realm are significant. Furthermore, they are even vital, especially in the analysis of dynamism, as a key factor in *deterring* the decline of psychological health. Accordingly, the intervention for fairness/justice with their ethical dimension is a protective way for psychological health.

The health inequality/disparity discussions often utilize the word ‘fair’ or ‘fair society: it is monumental that The Marmot Review in England, calling for social justice, was entitled Fair Society, Healthy Lives (2010) ([36,112]). These words signify ‘equal’ or ‘equal societies’ in most cases, and the former is, in fact, a synonym of the latter.

This study endorses this sense of fair society; at the same time, this illuminates that another aspect of fairness and justice is as vital as equality. While the former sense of fairness signifies (impartial) equality, the latter implies ethical uprightness or squareness, which corresponds to (ethical) fidelity to social practice ([113]): these two senses are, respectively, quantitative fairness (fairness as equality) and qualitative fairness (fairness as fidelity), and both composites equity (Equality and equity here correspond to arithmetic and geometrical equality/justice in Aristotle’s political philosophy. The concept of fairness is, in our view, four-dimensional, and there are two other kinds of fairness: (rule-)compliance(or law-abidingness: fairness as compliance) and (benefit) reciprocity(fairness as reciprocity) [114].).

Accordingly, reducing the multi-dimensional disparities into more equal situations would increase physical/psychological health and health equity, which is almost equivalent to health justice/fairness: this will lead to a fairer world. Furthermore, another ethico-political aspect of fairness/justice would also increase psychological health and health equity, bringing a world with more fairness/justice. Therefore, these recognitions may contribute to realizing a fair/just society across the globe.

## Figures and Tables

**Table 1:** Correlations between Psychological Health (PSH)/Mental Change/Feelings Changes and Basic Factors (Survey 1, 2).

		PSH			Mental Change	Increased Light Feelings			Increased Dark Feelings			Increased Anxiety			Increased Depression		
		Survey1 (N = 4698)	Survey2 (N = 6855)	Average	Survey1 (N = 4698)	Survey1 (N = 4698)	Survey2 (N = 6855)	Average	Survey1 (N = 4698)	Survey2 (N = 6855)	Average	Survey1 (N = 4698)	Survey2 (N = 6855)	Average	Survey1 (N = 4698)	Survey2 (N = 6855)	Average
		r			r	r											
Ascriptive factors(*4*)	Sex	0.053** [19]	0.006	△	0.059** [16]	0.025† [15]	−0.052** [12]	0.014 [12]	0043** [17]	0.072** [12]	0.058 [14]	0.109** [9]	0.089** [9]	0.099 [9]	0.083** [15]	0.099** [15]	0.091 [15]
	Age	0.033* [20]	0.124** [18]	0.079 [18]	0.048** [19]	−0.085** [1]	0.015	△	−0.035* [18]	−0.037** [19]	−0.036 [17]	0.084** [13]	0.033** [16]	0.058 [15]	0.038** [19]	−0.133** [12]	−0.047 [17]
	Occupation	0.239** [16]	0.194** [17]	0.217 [15]	−0.050** [18]	0.023	−0.022† [16]	△	0.003	−0.050** [16]	△	−0.032* [19]	−0.016	△	−0.050** [18]	−0.069** [18]	−0.060 [16]
	Marriage	0.161** [17]	0.231** [16]	0.196 [16]	−0.005	−0.044* [11]	−0.001	△	−0.025† [19]	−0.057** [14]	−0.041 [16]	0.016	−0.011	△	−0.023	−0.088** [17]	△
Biological factors(*3*/*2*)	Exercise/Foods	(0.466**)	0.705** [1]	(0.585) [4]	(−0.115**)	(0.051**)	0.073** [3]	(0.062) [6]	(−0.113**)	−0.098** [8]	(−0.106) [7]	(−0.123**)	−0.087** [10]	(−0.105) [7]	(−0.134**)	−0.155** [5]	(−0.145) [9]
	Exercise	0.375** [13]			−0.135** [3]	0.043** [12]			−0.110** [12]			−0.166** [1]			−0.159** [7]		
	Foods	0.556** [4]			−0.096** [11]	0.058** [7]			−0.115** [10]			−0.079** [14]			−0.110** [12]		
	Medical environment	0.480** [10]	0.565** [8]	0.523 [8]	−0.075** [13]	0.061** [5]	0.066** [7]	0.063 [5]	−0.112** [11]	−0.053** [15]	−0.083 [12]	−0.047** [16]	−0.044** [14]	−0.04 6[16]	−0.093** [13]	−0.125** [14]	−0.109 [13]
Natural and Cultural factors(*2*)	Natural environment	0.536** [8]	0.610** [5]	0.573 [5]	−0.061** [15]	0.042** [14]	0.058** [10]	0.050 [10]	−0.067** [15]	−0.042** [17]	−0.055 [15]	−0.036** [18]	−0.021† [17]	−0.028 [17]	−0.059** [16]	−0.134** [11]	−0.097 [14]
	Educational environment	0.549** [5]	0.684** [2]	0.617 [2]	−0.112** [9]	0.078** [2]	0.067** [6]	0.073 [1]	−0.124** [8]	−0.098** [7]	−0.111 [6]	−0.103** [10]	−0.077** [12]	−0.090 [10]	−0.144** [10]	−0.164** [4]	−0.154 [7]
Economic factors(*3*)	Income	0.539** [7]	0.591** [7]	0.565 [7]	−0.128** [6]	0.058** [7]	0.069** [4]	0.064 [4]	−0.141** [1]	−0.132** [2]	−0.137 [1]	−0.148** [5]	−0.145** [2]	−0.146 [2]	−0.167** [4]	−0.170** [3]	−0.168 [2]
	Assets	0.541** [6]	0.598** [6]	0.570 [6]	−0.135** [3]	0.066** [3]	0.069** [4]	0.065 [3]	−0.139** [3]	−0.129** [3]	−0.134 [2]	−0.147** [6]	−0.142** [3]	−0.145 [3]	−0.161** [6]	−0.174** [2]	−0.167 [3]
	Employment stability	0.462** [11]	0.553** [9]	0.508 [11]	−0.126** [7]	0.059** [6]	0.064** [8]	0.067 [2]	−0.125** [7]	−0.120** [5]	−0.123 [4]	−0.154** [4]	−0.130** [4]	−0.142 [4]	−0.167** [5]	−0.146** [8]	−0.156 [6]
Societal community factors(*4*)	Stratification satisfaction	0.692** [1]	0.638** [4]	0.665 [1]	−0.151** [1]	0.058** [7]	0.075** [2]	0.029 [11]	−0.141** [1]	−0.133** [1]	−0.137 [1]	−0.124** [8]	−0.120** [6]	−0.122 [6]	−0.157** [8]	−0.192** [1]	−0.175 [1]
	General trust	0.564** [2]	0.640** [3]	0.602 [3]	−0.103** [10]	0.042** [13]	0.061** [9]	0.052 [9]	−0.124* [9]	−0.067** [13]	−0.096 [9]	−0.091** [11]	−0.064** [13]	−0.078 [13]	−0.146** [9]	−0.154** [6]	−0.150 [8]
	Disparity recognition	0.086** [18]	0.239** [15]	0.163 [17]	0.053** [17]	0.014	0.020	△	0.062** [16]	0.092** [9]	0.077 [13]	0.067** [15]	0.099** [8]	0.083 [12]	0.055** [17]	0.026* [19]	0.041 [18]
	Disparity elimination	0.310** [15]	0.429** [12]	0.370 [13]	−0.095** [12]	0.022	0.049** [13]	△	−0.079** [14]	−0.090** [10]	−0.085 [11]	−0.091** [12]	−0.118** [7]	−0.104 [8]	−0.121** [11]	−0.142** [9]	−0.132 [10]
Political factors(*4*)	Fairness/Justice	0.419** [12]	0.365** [13]	0.392 [12]	−0.134** [5]	0.023	0.044** [14]	△	−0.135** [5]	−0.110** [6]	−0.123 [5]	−0.156** [3]	−0.129** [5]	−0.142 [4]	−0.188** [1]	−0.135** [10]	−0.161 [4]
	Anti−corruptive fairness	0.338** [14]	0.314** [14]	0.326 [14]	−0.141** [2]	−0.008	0.043** [15]	△	−0.132** [6]	−0.123** [4]	−0.128 [3]	−0.158** [2]	−0.169** [1]	−0.164 [1]	−0.184** [2]	−0.131** [13]	−0.158 [5]
	Human rights	0.560** [3]	0.483** [11]	0.522 [9]	−0.074** [14]	0.064** [4]	0.056** [11]	0.060 [8]	−0.105** [13]	−0.089** [11]	−0.097 [8]	−0.045** [17]	−0.083** [11]	−0.064 [14]	−0.085** [14]	−0.154** [6]	−0.119 [12]
	Civil efficiency	0.493** [9]	0.551** [10]	0.522 [9]	−0.122** [8]	0.045* [10]	0.077** [1]	0.061 [7]	−0.138** [4]	−0.039** [18]	−0.089 [10]	−0.132** [7]	−0.042** [15]	−0.087 [11]	−0.172** [3]	−0.090* *[16]	−0.131 [11]
	PSH	1.000	1.000	1.000	−0.157**	0.070**	0.105**	0.088	−0.177**	−0.140**	−0.159	−0.124**	−0.094**	−0.109	−0.200**	−0.226**	−0.213
	Increased light feelings	0.070**	0.105**	0.088	−0.133**	1.000	1.000	1.000	−0.067**	−0.077**	−0.072	−0.081**	−0.104**	−0.093	−0.069**	−0.092**	−0.081
	Increased dark feelings	−0.177**	−0.140**	−0.159	0.247**	−0.067**	−0.077**	−0.072	1.000	1.000	1.000	0.425**	0.294**	0.360	0.455**	0.309**	0.382
	Increased Anxiety	−0.124**	−0.094**	−0.109	0.237**	−0.081**	−0.104**	−0.092	0.425**	0.294**	0.360	1.000	1.000	1.000	0.432**	0.195**	0.314
	Increased Depression	−0.200**	−0.226**	−0.213	0.255**	−0.069**	−0.092**	−0.081	0.455**	0.309**	0.382	0.432**	0.195**	0.314	1.000	1.000	1.000
	Physical change	−0.145**			0.457**	−0.081**			0.140**			0.134**			0.169**		
	Mental change	−0.157**			1.000	−0.133**			0.247**			0.237**			0.255**		
	Fair society		0.428**				0.050**			−0.119**			−0.140**			−0.165**	
	Just society		0.426**				0.049**			−0.111**			−0.127**			−0.156**	
	Fair/Just society		0.243**				0.033**			−0.125**			−0.144**			−0.148**	
	Distributive justice		0.472**				0.057**			−0.117**			−0.150**			−0.168**	
	Contribution	0.505**	0.588**	0.547	−0.046*	0.045**	0.042**	0.044	−0.022	0.014		0.018	0.035*		−0.057**	−0.080**	−0.068
	Optimism	0.624**	0.752**	0.688	−0.145**	0.046**	0.100**	0.073	−0.168**	−0.135**	−0.152	−0.154**	−0.126**	−0.140	−0.204**	−0.206**	−0.205

Notes: ** *p* < 0.01 * *p* < 0.05 † *p* < 0.1. Sex (male = 0, female = 1). The figure for age is the actual age (non-logarithm). Occupation: no job = 0, otherwise = 1. Marital status: unmarried, divorced, or bereaved = 0, married = 1. The figures in parentheses concerning exercise/foods are based on the calculated values of correlation coefficients. The figure in brackets indicates the order (from the highest) of the magnitude concerning the estimated coefficient for each variable. The italicized figures in parentheses indicate the number of variables in each category; as for biological factors, refer to the below. △ shows no calculation is conducted because of the statistical non-significance (at 10% level) in Survey 1 or 2. The blank space indicates that the relevant variable is not asked in Survey 1 or Survey 2. “Exercise” and “Foods” are separately asked in Survey 1, while an integrated item, “Exercise/Foods”, is asked in Survey 2.

**Table 2:** Comparison of the Two Survey’s Correlation Coefficients in Each Category (PSH, change of feelings, and mental change).

	PSH			Mental Change	Increased Light Feelings			Increased Dark Feelings			Increased Anxiety			Increased Depression		
	Survey1	Survey2	Average	Survey1	Survey1	Survey2	Average	Survey1	Survey2	Average	Survey1	Survey2	Average	Survey1	Survey2	Average
Ascriptive factors(*4*)	0.122 (4.8%)	0.183 (6.2%)	0.153 (5.6%)	0.052 (8.4%)	0.051 (15.4%)	0.037 (10.5%)	0.044 (12.8%)	0.034 (5.6%)	0.054 (10.5%)	0.044 (7.9%)	0.075 (12.3%)	0.061 (11.7%)	0.068 (12.0%)	0.057 (7.9%)	0.097 (12.0%)	0.077 (10.1%)
	[6]	[6]	[6]	[6]	[5]	[6]	[6]	[6]	[6]	[6]	[5]	[5]	[5]	[6]	[6]	[6]
Biological factors(*3*/*2*)	0.470 (18.7%)	0.635 (21.4%)	0.553 (20.2%)	0.102 (16.5%)	0.054 (16.3%)	0.070 (19.8%)	0.062 (18.1%)	0.112 (18.5%)	0.076 (14.8%)	0.094 (16.8%)	0.097 (16.0%)	0.066 (12.7%)	0.082 (14.4%)	0.121 (16.8%)	0.140 (17.4%)	0.131 (17.1%)
	[3]	[2]	[2]	[4]	[4]	[1]	[4]	[3]	[4]	[4]	[3]	[4]	[4]	[3]	[3]	[3]
Natural and Cultural factors(*2*)	0.543 (21.6%)	0.647 (21.9%)	0.595 (21.7%)	0.117 (18.9%)	0.060 (18.1%)	0.063 (17.8%)	0.062 (18.1%)	0.096 (15.8%)	0.070 (13.6%)	0.083 (14.8%)	0.070 (11.5%)	0.049 (9.4%)	0.060 (10.5%)	0.102 (14.1%)	0.149 (18.5%)	0.126 (16.4%)
	[1]	[1]	[1]	[3]	[2]	[3]	[4]	[5]	[5]	[5]	[6]	[6]	[6]	[5]	[2]	[4]
Economic factors(*3*)	0.514 (20.4%)	0.581 (19.6%)	0.548 (20.0%)	0.130 (21.0%)	0.061 (18.4%)	0.067 (18.9%)	0.064 (18.7%)	0.135 (22.2%)	0.127 (24.8%)	0.131 (23.4%)	0.150 (24.7%)	0.139 (26.7%)	0.145 (25.6%)	0.165 (22.9%)	0.163 (20.2%)	0.164 (21.5%)
	[2]	[3]	[3]	[1]	[1]	[2]	[1]	[1]	[1]	[1]	[1]	[1]	[1]	[1]	[1]	[1]
Societal community factors(*4*)	0.413 (16.4%)	0.487 (16.4%)	0.450 (16.4%)	0.101 (16.3%)	0.050 (15.1%)	0.062 (17.5%)	0.056 (16.4%)	0.102 (16.8%)	0.096 (18.7%)	0.099 (17.7%)	0.093 (15.3%)	0.100 (19.2%)	0.097 (17.1%)	0.120 (16.6%)	0.129 (16.0%)	0.125 (16.3%)
	[5]	[4]	[4]	[5]	[6]	[4]	[2]	[4]	[2]	[3]	[4]	[3]	[3]	[4]	[4]	[5]
Political factors(*4*)	0.453 (18.0%)	0.428 (14.5%)	0.441 (16.1%)	0.118 (19.0%)	0.055 (16.6%)	0.055 (15.5%)	0.055 (16.1%)	0.128 (21.1%)	0.090 (17.5%)	0.109 (19.5%)	0.123 (20.2%)	0.106 (20.3%)	0.115 (20.3%)	0.157 (21.7%)	0.128 (15.9%)	0.143 (18.7%)
	[4]	[5]	[5]	[2]	[3]	[5]	[3]	[2]	[3]	[2]	[2]	[2]	[2]	[2]	[5]	[2]

Notes: The figures in parentheses indicate the share in the total correlation coefficients for categories. PSH: see Table 1. The italicized figures in parentheses indicate the number of variables in each category. The figure in brackets indicates the order (from the highest) concerning the magnitude of the estimated coefficient for each category.

**Table 3:** Multiple Regression Analysis of the Basic Factors for Surveys 1 and 2 (ranking of the top 10 items).

	PSH		Mental Change
	Survey1	Survey2	Survey1
R	0.815	0.835	0.203
R2	0.664	0.697	0.041
adjusted R2	0.663	0.696	0.040
1	Stratification	Exercise/Foods	Stratification
	(0.322**)	(0.296**)	(−0.087**)
2	Foods	Educational environment	Exercise
	(0.155**)	(0.149**)	(−0.060**)
3	General trust	General trust	Anti-corruptive fairness
	(0.126**)	(0.144**)	(−0.058**)
4	Natural environment	Stratification	Disparity recognition
	(0.114**)	(0.137**)	(0.053**)
5	Civil efficacy	Medical environment	Sex
	(0.114**)	(0.106**)	(0.043*)
6	Human rights	Natural environment	Employment stability
	(0.103**)	(0.088**)	(−0.039*)
7	Educational environment	Assets	Age
	(0.090**)	(0.046**)	(0.029*)
8	Medical environment	Civil efficacy	
	(0.078**)	(0.042**)	
9	Assets	Employment stability	
	(0.061**)	(0.036**)	
10	Marital status	Marital status	
	(0.052**)	(0.031**)	

Notes: ** *p* < 0.01 * *p* < 0.05. Independent variables: all basic factors, including ascriptive factors. Sex (male = 0, female = 1). The figure for age is the actual age (non-logarithm). Occupation: no job = 0, otherwise = 1. Marital status: unmarried, divorced, or bereaved = 0, married = 1. PSH and notes of other variables: see Table 1. Figures in parentheses denote standardized partial regression coefficients for each variable.

**Table 4:** Multiple Regression Analysis: Sum of Standardized Partial Regression Coefficients of the Basic Variables in Each Category.

	PSH			Mental Change
	Survey1	Survey2	Average	Survey1
Ascriptive factors(*4*)	0.149(11.4%)	0.081(6.8%)	0.115(9.2%)	0.072(19.5%)
	[5]*4*	[5]*3*	[5]	[2]*2*
Biological factors(*3*/*2*)	0.233(17.8%)	0.402(33.8%)	0.318(25.4%)	0.060(16.3%)
	[2]*2*	[1]*2*	[2]	[3]*1*
Natural and Cultural factors(*2*)	0.204(15.5%)	0.237(19.9%)	0.221(17.6%)	
	[3]*2*	[3]*2*	[3]	
Economic factors(*3*)	0.061(4.6%)	0.111(9.3%)	0.086(6.9%)	0.039(10.6%)
	[6]*1*	[4]*3*	[6]	[5]*1*
Societal community factors(*4*)	0.448(34.1%)	0.315(26.5%)	0.382(30.5%)	0.140(37.9%)
	[1]*2*	[2]*3*	[1]	[1]*2*
Political factors(*4*)	0.217(16.5%)	0.042(3.5%)	0.130(10.4%)	0.058(15.7%)
	[4]*2*	[6]*1*	[4]	[4]*1*

Notes: Independent variables: all basic factors, including ascriptive factors. Notes of other variables, including dummy variables: see Table 3. Total values of the partial regression coefficients (*β*) of all the variables within each category are listed (blank space for the value of 0). Figures in brackets indicate the order (from the highest) of the magnitude of the coefficient for each variable; as for ascriptive factors, coefficients alone are listed. The italicized figures in parentheses indicate the number of variables in each category. Figures in parentheses indicate the share in the total of standardized partial correlation coefficients for all categories. The italicized figures in the table indicate the order concerning the share in the column. Anti-corruptive fairness is not included in the calculation since the total of the category can become negative (due to the estimation results showing the reverse sign against the original theoretical conjecture).

**Table 5:** Results of Logistic Regression: Ranking of all Factors (Survey 1 and 2).

	Increased Light Feelings			Increased Dark Feelings			Increased Anxiety			Increased Depression		
	Survey1	Survey2_1	Survey2_2	Survey1	Survey2_1	Survey2_2	Survey1	Survey2_1	Survey2_2	Survey1	Survey2_1	Survey2_2
Cox-Snell R2	0.019	0.013	0.013	0.046	0.038	0.038	0.062	0.053	0.055	0.064	0.069	0.070
Nagelkerke R2	0.081	0.047	0.047	0.073	0.058	0.058	0.088	0.074	0.075	0.101	0.100	0.102
1	Educational environment	Civil efficacy	Civil efficacy	Disparity recognition	Disparity recognition	Disparity recognition	Employment stability	Anti-corruptive fairness	Anti-corruptive fairness	Employment stability	Stratification	Stratification
	(0.169*)	(0.133**)	(0.133**)	(0.101*)	(0.103**)	(0.103**)	(−0.076**)	(−0.103**)	(−0.087**)	(−0.082**)	(−0.098**)	(−0.101**)
2	Human rights	Stratification	Stratification	Civil efficacy	Stratification	Stratification	Disparity recognition	Disparity recognition	Income	Anti-corruptive fairness	Human rights	Distributive justice
	(0.141†)	(0.114**)	(0.114**)	(−0.088**)	(−0.086**)	(−0.086**)	(0.076	(0.070**)	(−0.084**)	(−0.078**)	(−0.071**)	(−0.087**)
3	Foods	Exercise/Foods	Exercise/Foods	Medical environment	Anti-corruptive fairness	Anti-corruptive fairness	Exercise	Income	Distributive justice	Fairness/Justice	Disparity recognition	Fair/just society
	(0.088†)	(0.096*)	(0.096*)	(−0.070**)	(−0.065**)	(−0.065**)	(−0.075**)	(−0.069**)	(−0.069**)	(−0.070*)	(0.056**)	(−0.058*)
4	Employment stability			Anti-corruptive fairness	Exercise/Foods	Exercise/Foods	Assets	Stratification	Stratification	Disparity recognition	Disparity elimination	Exercise/Foods
	(0.083†)			(−0.069**)	(−0.054**)	(−0.054**)	(−0.049*)	(−0.050**)	(−0.053**)	(0.064**)	(−0.056)	(−0.056**)
5	Anti-corruptive fairness			Income	Income	Income	Civil efficacy	Exercise/Foods	Disparity recognition	Civil efficacy	Exercise/Foods	Human rights
	(−0.181**)			(−0.066**)	(−0.051**)	(−0.084**)	(−0.043**)	(−0.047**)	(0.053**)	(−0.063**)	(−0.051**)	(−0.046*)
6				Stratification	Civil efficacy	Civil efficacy	Human rights	Assets	Exercise/Foods	Exercise	Assets	Assets
				(−0.052**)	(−0.045*)	(0.045*)	(0.058**)	(−0.040†)	(−0.048**)	(−0.060**)	(−0.045*)	(−0.044**)
7				Foods				Civil efficacy	Fair/just society	General trust	Educational environment	Disparity recognition
				(−0.041†)				(0.031†)	(−0.044†)	(−0.052*)	(−0.043†)	(0.031**)
8								Educational environment	Civil efficacy	Human rights	Civil efficacy	Civil efficacy
								(0.039†)	(0.049**)	(0.062**)	(0.058**)	(0.049*)
9								Natural environment	Educational environment			
								(0.044†)	(0.041†)			
10									Natural environment			
									(0.045*)			
**Ascriptive factors**												
1	Age	Occupation	Occupation	Occupation	Sex	Sex	Sex	Sex	Sex	Sex	Sex	Sex
	(−0.035**)	(−0.615**)	(−0.615**)	(0.538**)	(0.299**)	(0.299**)	(0.385**)	(0.427**)	(0.430**)	(0.315**)	(0.321**)	(0.305**)
2		Sex	Sex	Sex	Age	Age	Age	Age	Age		Occupation	Occupation
		(−0.606**)	(−0.606**)	(0.190*)	(−0.004†)	(−0.004†)	(0.007**)	(0.008**)	(0.008**)		(−0.206*)	(−0.214**)
3				Age							Age	Age
				(−0.008**)							(−0.019)	(−0.020**)

Notes: ** *p* < 0.01, * *p* < 0.05 † *p* < 0.1. Independent variables: all basic factors, including ascriptive factors. Notes of other variables, including dummy variables: see Table 3. The figures in parentheses denote partial regression coefficients. Those variables for which the results (sign) differ from the theoretical conjecture are below the dividing line (in ranking). As for ascriptive factors, the order is counted separately. Basic variables are calculated in Survey 1 and Survey 2_1 (Appendix F). In Survey 2_2, “Fair society”, “Just society”, “Fair/Just Society)”, and “Distribute justice” are additionally included in the calculation (Appendix G).

**Table 6:** Logistic Regression Analysis: Sum of Partial Regression Coefficients of the Basic Variables in each category.

	Increased Light Feelings			Increased Dark Feelings			Increased Anxiety			Increased Depression		
	Survey1	Survey2	Average	Survey1	Survey2	Average	Survey1	Survey2	Average	Survey1	Survey2	Average
Ascriptive factors(*4*)	0.035	1.221	0.628	0.736	0.303	0.520	0.392	0.435	0.414	0.315	0.546	0.431
											
Biological factors(*3*/*2*)	0.088 (18.3%)	0.096 (28.0%)	0.092 (22.3%)	0.111 (22.7%)	0.054 (15.0%)	0.083 (19.5%)	0.075 (19.9%)	0.047 (12.4%)	0.061 (16.1%)	0.060 (11.3%)	0.051 (12.5%)	0.056 (11.8%)
	[3]*1*	[3]*1*	[2]	[3]*2*	[3]*1*	[3]	[4]*1*	[4]*1*	[4]	[4]*1*	[3]*1*	[4]
Natural and Cultural factors(*2*)	0.169 (35.1%)		0.085 (20.5%)								0.043 (10.6%)	0.022 (4.6%)
	[1]*1*		[3]								[5]*1*	[5]
Economic factors(*3*)	0.083 (17.3%)		0.042 (10.1%)	0.066 (13.5%)	0.051 (14.2%)	0.059 (13.8%)	0.125 (33.2%)	0.109 (28.8%)	0.117 (31.0%)	0.082 (15.4%)	0.045 (11.1%)	0.064 (13.5%)
	[4]*1*		[5]	[4]*1*	[4]*1*	[4]	[1]*2*	[2]*2*	[1]	[3]*1*	[4]*1*	[3]
Societal community factors(*4*)		0.114 (33.2%)	0.057 (13.8%)	0.153 (31.4%)	0.189 (52.6%)	0.171 (40.4%)	0.076 (20.2%)	0.120 (31.7%)	0.098 (25.9%)	0.116 (21.8%)	0.210 (51.6%)	0.163 (34.8%)
		[2]*1*	[4]	[2]*2*	[1]*2*	[1]	[3]*1*	[1]*2*	[3]	[2]*2*	[1]*3*	[2]
Political factors(*4*)	0.141 (29.3%)	0.133 (38.8%)	0.137 (33.3%)	0.157 (32.0%)	0.065 (18.1%)	0.111 (26.2%)	0.101 (26.8%)	0.103 (27.2%)	0.102 (27.0%)	0.273 (51.4%)	0.058 (14.3%)	0.166 (35.3%)
	[2]*1*	[1]*1*	[1]	[1]*2*	[2]*1*	[2]	[2]*2*	[3]*1*	[2]	[1]*4*	[2]*1*	[1]

Notes: Independent variables: all basic factors, including ascriptive factors. Notes of other variables, including dummy variables: see Table 3. Total values of the partial regression coefficients (β) of all the variables within each category are listed (blank space for the value of 0). The figures in brackets indicate the order (from the highest) of the total magnitude concerning the coefficients in each category. Ascriptive factors*’* share and order are not calculated, and their total values of partial regression coefficients alone are listed. The italicized figures in parentheses indicate the number of variables in each category. The figures in parentheses indicate the share in the total of coefficients for all categories. The italicized figures in the table indicate the order concerning the share in the column. Variables in the estimation results with the reverse sign against the theoretical conjecture (Anti-corruptive fairness for *“*Increased light feelings*”* in Survey 1; Civil efficacy for *“*Increased dark feelings*”*, Natural environment, Educational environment and Civil efficacy for *“*Increased anxiety*”*, and Human rights for *“*Increased depression*”* in Survey 2) are not included in the calculation so that the total of the category cannot become negative.

## Data Availability

As for the data used in this study, please contact the corresponding author (Masaya Kobayashi).

## Appendix A. Questions in the Three Surveys

### Appendix A.1. Factors in Survey 1 and Survey 2

**Category**	**Factor**	**Survey 1**	**Survey 2**	**Answer**
Ascriptive factors	sex	Please let us know your sex.	Please let us know your sex.	1 (Male), 2 (Female)
	age	Please let us know your age.	Please let us know your age.	
	occupation	Please let us know your occupation.	Please let us know your occupation.	See Appendix B, “Occupation”
	marriage	Are you married?	Are you married?	See Appendix B, “Marital status”
Biological factors	exercise/foods		Do you think you are doing healthy exercise and eating?	1 = not at all, 10 = very much
	exercise	Do you consider your exercise habits to be adequate?		
	foods	Do you consider yourself to eat healthily?		
	medical environment	Do you think the medical environment in your neighborhood, such as hospitals and pharmacies, is well-developed?	Do you think the medical environment in your neighborhood, such as hospitals and pharmacies, is well-developed?	
Natural and Cultural factors	natural environment	How rich and blessed do you feel about the natural environment surrounding you?	Do you think the natural environment surrounding you is good?	1 = not at all, 10 = very much
	educational environment	How well do you feel about your own educational or lifelong learning environment and the learning environment of the children around you?	Do you think your own educational or lifelong learning and the learning environment of children around you are fulfilling?	
Economic factors	income	Do you think your income is sufficient for you to make a living now that COVID-19 has struck?	Do you think your income is sufficient to live your life?	1 = not at all, 10 = very much
	assets	Do you think you have sufficient assets (financial, house, land, car, etc.) to live your life now that COVID-19 has occurred?	Do you consider your assets (financial, house, land, car, etc.) sufficient for your life?	
	employment stability	Now that COVID-19 has occurred, do you consider your employment to be stable?	Do you feel that you have stability in your employment?	
Societal community factors	stratification satisfaction	I think I am satisfied with my social status and stratification.	Are you satisfied with your social status and stratification?	1 = not at all, 10 = very much
	general trust	Do you find people generally trustworthy?	Do you find people generally trustworthy?	
	disparity recognition	How much disparity do you think exists in the society around you?	Do you think that there is a disparity in the society around you?	
	disparity elimination	Do you think that the society around you realizes the elimination of disparities (equal society) through social welfare and redistribution through taxes?	Do you think that the society around you realizes the elimination of disparity (equal society) through social welfare and redistribution through taxes?	
Political factors	fairness/justice	I believe that fairness and justice are achieved in our country’s politics in terms of decision-making, the disparity between rich and poor, and so on.	Do you think that Japanese politics achieve fairness and justice in terms of decision-making, the disparity between rich and poor, and so on?	1 = not at all, 10 = very much
	Anti-corruptive fairness	I think that my government is corruption-free and fair.	Do you think that the Japanese government is corruption-free and fair?	
	human rights	I believe that fundamental human rights are respected in my country.	Do you think that fundamental human rights are respected in Japan?	
	civil efficacy	How much do you think you can change the society and politics around you in a desirable direction through your involvement?	Do you want to change the society and politics around you in a desirable direction through your involvement?	

Additional Factors

**Contribution Optimism**	**contribution**	**Do you want to contribute to society?**	**0 = not at all,** **10 = completely**
	optimism	How optimistic would you say you are about your future?	
Fair society Just society	fair society	All things to be considered, I think our current society is fair.	0 = not at all, 10 = completely
	just society	All things to be considered, I think our current society is just.	
Fair/Just Society *	1	All things to be considered, I think our current society is fair.	0 = not at all, 10 = completely
	2	All things to be considered, I think our current society is unfair.	
	3	All things to be considered, I think our current society is just.	
	4	All things to be considered, I think our current society is unjust.	
Distributive Justice **	disparity of justice	Do you think the disparity in Japan is in the right/just state?	0 = not at all, 10 = completely
	welfare justice	Do you think that welfare is rightly/justly correcting the disparity in current society?	
Made by the authors. Notes: * The score for Fair society/Just society is calculated as the “sum of the scores for the items 1, 2, 3 and 4” divided by 4. The score for item 2 and item 4 is calculated by the subtraction of 11 from the original figure. ** As for Distributive Justice, the score is calculated by “the sum of the scores for the disparity of justice, welfare justice, and disparity elimination” divided by 3.			

### Appendix A.2. Changes

Mental Changes (Survey 1)

What changes have you seen in your own situation from March (2020), when Covid-19 became more serious in Japan, until now?

**Item**	**Survey 1**
**Mental change**	Mental changes, such as anxiety and restlessness.

Method: The scale of responses is as follows.

Response 1: Very much better/very much stronger

Response 2: Slightly better/slightly stronger

Response 3: Not much change

Response 4: Slightly worse/slightly weaker

Response 5: Very much worse/very much weaker

Feelings Changes (Survey 1 & 2)

Mental condition from last March (2020), when COVID-19 became serious in Japan, until now.

**Item**	**Survey 1·2**
**Increased light feelings**	My mental state was lighter than usual.
**Increased dark feelings**	My mental state was darker than usual.
**Increased anxiety**	My mental state was more anxious than usual.
**Increased depression**	My mental state was more depressed than usual.
Note: For both Survey 1 and Survey 2, the multiple-choice from the ten items is applied. The table above lists the variables used in this paper alone.	

### Appendix A.3. PERMA Profiler, SWLS, I COPPE, and Revised HEMA—R

SWLS

Below are five statements that you may agree or disagree with. Indicate your agreement with each item by placing the appropriate number on the line preceding that item. Please be open and honest in your response.

	**Question**	**Answer in this survey**	**Original answer**
**1**	**In most ways, my life is close to my ideal.**	1 = Strongly disagree, 10 = Strongly agree	1 = strongly disagree, 2 = Disagree, 3 = Slightly disagree, 4 = Neither agree nor disagree, 5 = Slightly agree, 6 = Agree, 7 = Strongly agree
**2**	**The conditions of my life are excellent.**		
**3**	**I am satisfied with my life.**		
**4**	**So far, I have gotten the important things I want in life.**		
**5**	**If I could live my life over, I would change almost nothing.**		
Notes: For details, see the site on SWLS (http://labs.psychology.illinois.edu/~ediener/SWLS.html, accessed on 19 November 2022).		

PERMA Profiler

**#**	**Label**	**Question**	**Answer in This Survey**	**Original Response Anchors**
Block 1	A1	How much of the time do you feel you are making progress toward accomplishing your goals?	1 = not at all, 10 = completely	0 = never, 10 = always
	E1	How often do you become absorbed in what you are doing?		
	P1	In general, how often do you feel joyful?		
	N1	In general, how often do you feel anxious?		
	A2	How often do you achieve the important goals you have set for yourself?		
Block 2	H1	In general, how would you say your health is?		0 = terrible, 10 = excellent
Block 3	M1	In general, to what extent do you lead a purposeful and meaningful life?		0 = not at all, 10 = completely
	R1	To what extent do you receive help and support from others when you need it?		
	M2	In general, to what extent do you feel that what you do in your life is valuable and worthwhile?		
	E2	In general, to what extent do you feel excited and interested in things?		
	Lon	How lonely do you feel in your daily life?		
Block 4	H2	How satisfied are you with your current physical health?		0 = not at all, 10 = completely
Block 5	P2	In general, how often do you feel positive?		0 = never, 10 = always
	N2	In general, how often do you feel angry?		
	A3	How often are you able to handle your responsibilities?		
	N3	In general, how often do you feel sad?		
	E3	How often do you lose track of time while doing something you enjoy?		
Block 6	H3	Compared to others of your same age and sex, how is your health?		0 = terrible, 10 = excellent
Block 7	R2	To what extent do you feel loved?		0 = not at all, 10 = completely
	M3	To what extent do you generally feel you have a sense of direction in your life?		
	R3	How satisfied are you with your personal relationships?		
	P3	In general, to what extent do you feel contented?		
Block 8	hap	Taking all things together, how happy would you say you are?		0 = not at all, 10 = completely
Notes: For details, see [95]. P = Positive emotions, E = Engagement, R = Relationships, M = Meaning, A = Accomplishment, H = Health, N = Negative emotions, Lon = Lonely, hap = happiness.				

I COPPE

All questions start with the following stem: The top number ten represents the best your life can be. The bottom number zero represents the worst your life can be. When it comes to….

**Label**	**Question**	**Answer in This Survey**	**Original Answer**
**OV_WB_PR**	When it comes to the best possible life for you, on which number, do you stand now?	1 = the worst your life can be 10 = the best your life can be	0 = the worst your life can be 10 = the best your life can be
**OV_WB_PA**	When it comes to the best possible life for you, on which number, did you stand five years ago?		
**OV_WB_FU**	When it comes to the best possible life for you, on which number, do you think you will stand five years from now?		
**IN_WB_PR**	When it comes to relationships with important people in your life, on which number, do you stand now?	1 = the worst your life can be 10 = the best your life can be	0 = the worst your life can be 10 = the best your life can be
**IN_WB_FU**	When it comes to relationships with important people in your life, on which number, do you think you will stand five years from now?		
**CO_WB_PR**	When it comes to the community where you live, on which number, do you stand now?	1 = the worst your life can be 10 = the best your life can be	0 = the worst your life can be 10 = the best your life can be
**CO_WB_FU**	When it comes to the community where you live, on which number, do you think you will stand five years from now?		
**OC_WB_PR**	When it comes to your main occupation (employed, self-employed, volunteer, stay at home), on which number, do you stand now?	1 = the worst your life can be 10 = the best your life can be	0 = the worst your life can be 10 = the best your life can be
**OC_WB_FU**	When it comes to your main occupation (employed, self-employed, volunteer, stay at home), on which number, do you think you will stand five years from now?		
**PH_WB_PR**	When it comes to your physical health, on which number, do you stand now?	1 = the worst your life can be 10 = the best your life can be	0 = the worst your life can be 10 = the best your life can be
**PH_WB_FU**	When it comes to your physical health, on which number, do you think you will stand five years from now?		
**PS_WB_PR**	When it comes to your emotional and psychological well-being, on which number, do you stand now?	1 = the worst your life can be 10 = the best your life can be	0 = the worst your life can be 10 = the best your life can be
**PS_WB_FU**	When it comes to your emotional and psychological well-being, on which number, do you think you will stand five years from now?		
**EC_WB_PR**	When it comes to your economic situation, on which number, do you stand now?	1 = the worst your life can be 10 = the best your life can be	0 = the worst your life can be 10 = the best your life can be
**EC_WB_FU**	When it comes to your economic situation, on which number, do you think you will stand five years from now?		
Notes: For details, see [96]. OV_WB = Overall Well-Being, IN_WB = Interpersonal Well-Being, CO_WB = Community Well-Being, OC_WB = Occupational Well-Being, PH_WB = Physical Well-Being, PS_WB = Psychological Well-Being, EC_WB = Economic Well-Being. PR = Present, PA = Past, FU = Future. In the original survey, the treatment of PA (Past) is applied to variables from IN_WB to EC_WB; in our survey, this was not applied. In addition, there was OV_WB_PA in our survey, but this study did not use the question. In the original survey (above), PA (Past) denotes “a year ago”, and FU(Future) denotes “a year from now”; in our survey, these are modified to “five years ago” and “five years from now”, respectively. The reason for this modification (from one year to five years) is to ensure that respondents consider their situation well before (for the case of PA) or well after (for the case of FU) the outbreak of COVID-19.			

Revised HEMA—R

To what degree do you typically approach your activities with each of the following intentions, whether or not you actually achieve your aim?

	**Question**	**Answer in This Survey**	**Original answer**
**1**	Seeking relaxation?	1 = not at all, 10 = very much	1 = not at all, 7 = very much
**2**	Seeking to develop a skill, learn, or gain insight into something?		
**3**	Seeking to do what you believe in?		
**4**	Seeking pleasure?		
**5**	Seeking to pursue excellence or a personal ideal?		
**6**	Seeking enjoyment?		
**7**	Seeking to take it easy?		
**8**	Seeking to use the best in yourself?		
**9**	Seeking fun?		
**10**	Seeking to contribute to others or the surrounding world?		
Notes: For details, see [104]. In this survey, the response scale of 1 (not at all) to 10 (very much) is adopted.			

## Appendix B. Respondents of the Three Surveys (after Data Screening)

	**Survey 1 (%)**	**Survey 2 (%)**	**Survey 3 (%)**
Number of respondents	4698	6855	2472
Number of survey questions	383	401	174
**Residence**			
16 prefectures with big cities	2783(59.2)	1520(22.2)	1207(48.8)
32 prefectures without big cities	1915(40.8)	5335(77.8)	1265(51.2)
**Sex**			
Male	2283(48.6)	4404(64.2)	1626(65.8)
Female	2415(51.4)	2451(35.8)	846(34.2)
**Age**			
10’s	790(16.8)	36(0.5)	7(0.3)
20’s	759(16.2)	460(6.7)	125(5.1)
30’s	785(16.7)	1038(15.1)	346(14.0)
40’s	783(16.7)	1726(25.2)	610(24.7)
50’s	777(16.5)	1740(25.4)	626(25.3)
60’s	804(17.1)	1236(18.0)	480(19.4)
70’s and more		619(9.0)	278(11.2)
**Marital status**			
married	2172(46.2)	4074(59.4)	1418(57.4)
unmarried	2301(49.0)	2242(32.7)	846(34.2)
separation	225(4.8)	539(7.9) *	208(8.4) **
**Occupation**			
executive of a company or association	44(0.9)	123(1.8)	53(2.1)
office worker, staff of an association	1386(29.5)	2085(30.4)	734(29.7)
Part-time employee, contract employee, dispatched labor	206(4.4)	1196(17.4)	433(17.5)
Part-time worker, part-time job, home-based workers without an employment contract	585(12.5)	17(0.2)	7(0.3)
civil servants	140(3.0)	253(3.7)	68(2.8)
Self-employed, family employee, freelance	286(6.1)	818(11.9)	294(11.9)
faculty member		123(1.8)	39(1.6)
student	795(16.9)	95(1.4)	26(1.1)
homemaker	700(14.9)	766(11.2)	292(11.8)
pensioner	147(3.1)	603(8.8)	267(10.8)
none	365(7.8)	690(10.1)	240(9.7)
others	44(0.9)	86(1.3)	19(0.8)
**Education**			
currently attending high school	351(7.5)	43(0.6)	7(0.3)
currently attending vocational college, specialized training college	75(1.6)	84(1.2)	26(1.1)
currently attending junior college, college	48(1.0)	47(0.7)	8(0.3)
university/college preparatory school	14(0.3)	4(0.1)	
currently attending university	366(7.8)	88(1.3)	35(1.4)
currently attending a Master’s or Doctoral course	22(0.5)	19(0.3)	3(0.1)
junior high school	70(1.5)	175(2.6)	50(2.0)
high school	997(21.2)	2153(31.4)	664(26.9)
vocational college, specialized training college	370(7.9)	638(9.3)	240(9.7)
junior college, college	404(8.6)	598(8.7)	217(8.8)
university	1778(37.8)	2657(38.8)	1081(43.7)
more than a Master’s degree	203(4.3)	349(5.1)	141(5.7)
Notes: * divorce 418 (6.1)/death 121 (1.8). ** divorce 161 (6.5)/death 47 (1.9).			

## Appendix C. Situation of COVID-19 in Japan (January 2020-April 2022)

**Day/Month/Year**	**The Situation in Japan and in the World Where Relevant**	
**14/01/2020**	WHO confirmed the new corona disease.	
**15/01/2020**	Infection was first confirmed in Japan.	
**31/01/2020**	The government designated the infectious disease caused by the new coronavirus as a “designated infectious disease”.	
**03/02/2020**	The cruise ship “Diamond Princess”, with confirmed passenger infection, arrived at Yokohama Port.	
**27/02/2020**	Prime Minister Abe announced the intention to request the temporary closure of all elementary, junior high, and high schools nationwide.	
**28/02/2020**	The Governor of Hokkaido independently issued a “State of Emergency”.	
**10/03/2020**	The government designated the coronavirus situation as a “historical emergency” for the first time.	1st wave
**11/03/2020**	WHO declared a “pandemic”.	
**24/03/2020**	It was decided to postpone the Tokyo Olympics and Paralympics for about one year.	
**01/04/2020**	Prime Minister Abe announced a policy of distributing two cloth masks to households nationwide.	
**07/04/2020**	The “State of Emergency”(1st) based on the Act on Special Measures against the New Coronavirus was issued to the seven prefectures of Tokyo, Kanagawa, Saitama, Chiba, Osaka, Hyogo, and Fukuoka. (The declaration was effective until 6 May 2020.)	
**16/04/2020**	The area subject to a “State of Emergency” was expanded to the whole country (the declaration was to take effect until 6 May).	
	Thirteen prefectures (Tokyo, Kanagawa, Saitama, Chiba, Osaka, Hyogo, Fukuoka, Hokkaido, Ibaraki, Ishikawa, Gifu, Aichi, and Kyoto) were designated as “Special Warning Prefectures”.	
**16/04/2020**	Prime Minister Abe announced his intention to provide 100,000 yen per person to all citizens.	
**18/04/2020**	The number of infected people in Japan exceeded 10,000.	
**04/05/2020**	It was officially decided to extend the “State of Emergency“ until 31 May while keeping the area subject to it nationwide.	
**14/05/2020**	The “State of Emergency“ was lifted in 39 prefectures.	
	Eight prefectures of Hokkaido, Tokyo, Kanagawa, Chiba, Saitama, Osaka, Hyogo, and Kyoto continued to declare a “State of Emergency.“	
**25/05/2020**	The State of Emergency was lifted nationwide.	
**02/06/2020**	The first survey started.	
**04/06/2020**	The first survey was completed.	
**16/07/2020**	The daily number of infected people in Japan exceeded 600.	2nd wave
**26/07/2020**	The total number of infected people in Japan exceeded 30,000.	
**11/08/2020**	The total number of infected people in Japan exceeded 50,000.	
**16/09/2020**	The Abe Cabinet resigned, and the new Suga Cabinet was formed.	
**07/01/2021**	Tokyo, Saitama, Chiba, and Kanagawa declared a “State of Emergency“(2nd) (for the period until 7 February).	3rd wave
**13/01/2021**	In addition to Tokyo and its neighboring three prefectures (Kanagawa, Saitama, and Chiba), The “State of Emergency“ was declared in seven prefectures: Osaka, Hyogo, Kyoto, Aichi, Gifu, Fukuoka, and Tochigi.	
	It was decided to suspend the entry of foreigners completely.	
**17/02/2021**	Advanced vaccination of the new coronavirus vaccine began for medical workers.	
**26/02/2021**	The “State of Emergency“ was lifted in six prefectures: Osaka, Hyogo, Kyoto, Aichi, Gifu, and Fukuoka.	
**21/03/2021**	The “State of Emergency” in Tokyo, Saitama, Chiba, and Kanagawa was lifted.	4th wave
**24/03/2021**	The second survey started.	
**25/03/2021**	The second survey was completed.	
**05/04/2021**	The three prefectures of Osaka, Hyogo, and Miyagi were subjected to “Priority Measures to Prevent the Spread of Disease” for one month, from 5 April to 5 May.	
**12/04/2021**	Corona vaccination for the elderly began.	
	The “Priority Measures to Prevent the Spread of Disease” was applied to the three prefectures of Tokyo, Kyoto, and Okinawa (until 5 May in Kyoto and Okinawa, and until 11 May in Tokyo).	
**20/04/2021**	The four prefectures of Saitama, Chiba, Kanagawa, and Aichi were subjected to “Priority Measures to Prevent the Spread of Disease”.	
**23/04/2021**	The “Priority Measures to Prevent the Spread of Disease” was applied to Ehime.	
**25/04/2021**	The “State of Emergency“ (3rd) was declared in Tokyo, Osaka, Hyogo, and Kyoto (for the period until May 11).	
**07/05/2021**	It was decided to extend the “State of Emergency” to the four prefectures of Tokyo, Osaka, Hyogo, and Kyoto until 31 May.	
**09/05/2021**	The three prefectures of Hokkaido, Gifu, and Mie were subjected to “Priority Measures to Prevent the Spread of Disease”.	
**12/05/2021**	Aichi and Fukuoka declared a “State of Emergency.“	
**16/05/2021**	Hokkaido, Okayama, and Hiroshima declared a “State of Emergency.“	
	The three prefectures of Gunma, Ishikawa, and Kumamoto were subjected to “Priority Measures to Prevent the Spread of the Disease”.	
**23/05/2021**	A “State of Emergency“ was declared in Okinawa (for the period until 20 June).	
**28/05/2021**	It was decided to extend the deadline of 31 May for the “State of Emergency” declared in the nine prefectures of Hokkaido, Tokyo, Aichi, Osaka, Hyogo, Kyoto, Okayama, Hiroshima, and Fukuoka to 20 June.	
	It was decided to extend the deadline for “Priority Measures to Prevent the Spread of Disease” in the five prefectures of Saitama, Chiba, Kanagawa, Gifu, and Mie until 20 June.	
**20/06/2021**	The “State of Emergency” issued to 10 prefectures was lifted in 9 prefectures except for Okinawa.	
	The seven prefectures of Hokkaido, Tokyo, Aichi, Osaka, Hyogo, Kyoto, and Fukuoka shifted from a “State of Emergency“ to“ priority measures to prevent the spread of the disease“ (for the period until 11 July.)	
	Priority Measures to Prevent the Spread of Disease in Gifu and Mie were lifted (Saitama, Chiba, and Kanagawa prefectures extended the period until 11 July).	
**12/07/2021**	The “State of Emergency“(4th) was declared for Tokyo (for the period until 22 August).	5th wave
	The “State of Emergency” in Okinawa was extended until 22 August.	
	The “Priority Measures to Prevent the Spread of the Disease” in Saitama, Chiba, Kanagawa, and Osaka were extended until 22 August (lifted in Hokkaido, Aichi, Kyoto, Hyogo, and Fukuoka).	
**23/07/2021**	The Tokyo Olympics began.	
**02/08/2021**	In addition to Tokyo and Okinawa, a “State of Emergency“ was declared in Saitama, Chiba, Kanagawa, and Osaka (for the period until 31 August).	
	Five prefectures of Hokkaido, Ishikawa, Hyogo, Kyoto, and Fukuoka were subjected to “Priority Measures to Prevent the Spread of Disease“ (for the period until 31 August).	
**08/08/2021**	The Tokyo Olympics came to a close.	
	The eight prefectures of Fukushima, Ibaraki, Tochigi, Gunma, Shizuoka, Aichi, Shiga, and Kumamoto were subjected to “Priority Measures to Prevent the Spread of Disease” (for the period until 31 August).	
**20/08/2021**	The “State of Emergency“ was declared in seven prefectures: Ibaraki, Tochigi, Gunma, Shizuoka, Kyoto, Hyogo, and Fukuoka. In addition, Tokyo, Okinawa, Saitama, Chiba, Kanagawa, and Osaka have been extended (the new period was until 12 September).	
	“Priority Measures to Prevent the Spread of Disease” was applied to 10 prefectures: Miyagi, Yamanashi, Toyama, Gifu, Mie, Okayama, Hiroshima, Kagawa, Ehime, and Kagoshima (for the period until 12 September).	
**24/08/2021**	The Tokyo Paralympic Games began.	
**27/08/2021**	The “State of Emergency“ was declared in Hokkaido, Miyagi, Gifu, Aichi, Mie, Shiga, Okayama, and Hiroshima (for the period until 12 September).	
	The four prefectures of Kochi, Saga, Nagasaki, and Miyazaki were subjected to “Priority Measures to Prevent the Spread of Disease” (for the period until 12 September).	
**05/09/2021**	The Tokyo Paralympic Games came to a close.	
**13/09/2021**	The “State of Emergency“ in 19 prefectures, including Tokyo and Osaka, was extended until 30 September. Miyagi Prefecture and Okayama Prefecture shifted to “Priority Measures to Prevent the Spread of the Disease. “	
	The six prefectures of Toyama, Yamanashi, Ehime, Kochi, Saga, and Nagasaki lifted their “Priority Measures to Prevent the Spread of Disease”.	
	“Priority Measures to Prevent the Spread of the Disease“ in Miyagi, Fukushima, Ishikawa, Okayama, Kagawa, Miyazaki, Kumamoto, and Kagoshima were extended until 30 September.	
**28/09/2021**	The government decided to lift all 19 prefectures’ “State of Emergency” and eight prefectures’ “Priority Measures to Prevent the Spread of Disease” on 30 September.	
**30/09/2021**	The “State of Emergency” of 19 prefectures and the “Priority Measures to Prevent the Spread of Disease” of 8 prefectures were lifted.	
**04/10/2021**	The Suga cabinet resigned, and the new Kishida cabinet was formed.	
**26/10/2021**	The number of people who received the second dose of the new coronavirus vaccine exceeded 70% of the total population.	
	The third survey started.	
**28/10/2021**	The third survey was completed.	
**01/12/2021**	The third round of new coronavirus vaccination began nationwide for medical workers.	6th wave
**09/01/2022**	“Priority Measures to Prevent the Spread of the Disease“ were applied to the three prefectures of Okinawa, Yamaguchi, and Hiroshima (for the period until 31 January).	
**21/01/2022**	“Priority Measures to Prevent the Spread of Disease“ were applied to 13 prefectures of Tokyo, Saitama, Chiba, Kanagawa, Gunma, Niigata, Aichi, Gifu, Mie, Kagawa, Nagasaki, Kumamoto, and Miyazaki (for the period until 13 February).	
**27/01/2022**	“Priority Measures to Prevent the Spread of Disease” were applied to 18 prefectures: Hokkaido, Aomori, Yamagata, Fukushima, Ibaraki, Tochigi, Ishikawa, Nagano, Shizuoka, Kyoto, Osaka, Hyogo, Shimane, Okayama, Fukuoka, Saga, Oita, and Kagoshima (for the period until 20 February).	
	The period for the “Priority Measures to Prevent the Spread of Disease” in Okinawa, Yamaguchi, and Hiroshima was extended to 20 February.	
**05/02/2022**	“Priority Measures to Prevent the Spread of Disease” was applied to Wakayama (for the period until 27 February 2022).	
**12/02/2022**	“Priority Measures to Prevent the Spread of the Disease“ were applied to Kochi (for the period until 6 March).	
**13/02/2022**	“Priority Measures to Prevent the Spread of the Disease“ in Tokyo, Saitama, Chiba, Kanagawa, Gunma, Niigata, Aichi, Gifu, Mie, Kagawa, Nagasaki, Kumamoto, and Miyazaki were extended until 6 March.	
**20/02/2022**	“Priority Measures to Prevent the Spread of Disease“ in Hokkaido, Aomori, Fukushima, Ibaraki, Tochigi, Ishikawa, Nagano, Shizuoka, Kyoto, Osaka, Hyogo, Okayama, Hiroshima, Fukuoka, Saga, and Kagoshima were extended until 6 March.	
	“Priority Measures to Prevent the Spread of Disease” were lifted in Okinawa, Yamagata, Shimane, Yamaguchi, and Oita.	
**06/03/2022**	“Priority Measures to Prevent the Spread of Disease” in Hokkaido, Aomori, Ibaraki, Tochigi, Gunma, Saitama, Chiba, Tokyo, Kanagawa, Ishikawa, Gifu, Shizuoka, Aichi, Kyoto, Osaka, Hyogo, Kagawa, and Kumamoto were extended until 21 March.	
	“Priority Measures to Prevent the Spread of Disease“ in Fukushima, Niigata, Nagano, Mie, Wakayama, Okayama, Hiroshima, Kochi, Fukuoka, Saga, Nagasaki, Miyazaki, and Kagoshima were lifted.	
**22/03/2022**	“Priority Measures to Prevent the Spread of Disease“ in Hokkaido, Aomori, Ibaraki, Tochigi, Gunma, Saitama, Chiba, Tokyo, Kanagawa, Ishikawa, Gifu, Shizuoka, Aichi, Kyoto, Osaka, Hyogo, Kagawa, and Kumamoto were lifted.	
Notes: The content of “State of Emergency” includes requests for cooperation in refraining from going out and restricting the use of facilities, as well as requests for cooperation necessary to prevent infection. The content of “Priority Measures to Prevent the Spread of Diseases” includes requests to restaurants to shorten their working hours until 8 p.m. and to inform customers of infection prevention measures such as wearing masks and prohibiting entry by those who do not comply with such measures, as well as requests to residents not to visit restaurants unnecessarily during the restricted hours. Source: Based on NHK Special site on New Corona Virus (https://www3.nhk.or.jp/news/special/coronavirus/chronology/, accessed on 19 November 2022).		

## Appendix D. Multiple Regression Analysis of the Basic Factors (Surveys 1 and 2)

		**PSH**		**Mental Change**
		**Survey1**	**Survey2**	**Survey1**
	R	0.815	0.835	0.203
	R2	0.664	0.697	0.041
	adjusted R2	0.663	0.696	0.040
		***β***		
Ascriptive factors(*4*)	Sex	0.031**		0.043**
		[13]		[5]
	Age	−0.033*	0.023**	0.029*
		[11]	[13]	[7]
	Occupation	0.033**	0.027**	
		[11]	[12]	
	Marriage	0.052**	0.031**	
		[10]	[10]	
Biological factors(*3*/*2*)	Exercise/Foods	-	0.296**	
		-	[1]	
	Exercise		-	−0.060**
			-	[2]
	Foods	0.155**	-	
		[2]	-	
	Medical environment	0.078**	0.106**	
		[8]	[5]	
Natural and Cultural factors(*2*)	Natural environment	0.114**	0.088**	
		[4]	[6]	
	Educational environment	0.090**	0.149**	
		[7]	[2]	
Economic factors(*3*)	Income		0.029*	
			[11]	
	Assets	0.061**	0.046**	
		[9]	[7]	
	Employment stability		0.036**	−0.039*
			[9]	[6]
Societal community factors(*4*)	Stratification satisfaction	0.322**	0.137**	−0.087**
		[1]	[4]	[1]
	General trust	0.126**	0.144**	
		[3]	[3]	
	Disparity recognition		0.034**	0.053**
			[14]	[4]
	Disparity elimination			
		
Political factors(*4*)	Fairness/Justice			
		
	Anti-corruptive fairness	−0.076**	−0.048**	−0.058**
		[14]	[15]	[3]
	Human rights	0.103**		
		[6]		
	Civil efficiency	0.114**	0.042**	
		[4]	[8]	
Notes: ** *p* < 0.01, * *p* < 0.05. Independent variables: all basic factors, including Ascriptive factors. PSH and notes of other variables: see Table 1. Blank spaces indicate that the factor does not appear in the analysis. The figure in brackets indicates the order (from the highest) of the magnitude of each variable. “Exercise” and “Foods” are separately asked in Survey 1, while an integrated item, “exercise/Foods”, is asked in Survey 2. - indicates ‘no calculation.’ The italicized figures in parentheses indicate the number of variables in each category; as for biological factors, refer to Table 1.				

## Appendix E. Regression Analysis: Standardized Partial Regression Coefficients of the Basic Factors (Surveys 1 and 2)

### Appendix E.1. Multiple Regression Analysis: Total Value of the Standardized Partial Regression Coefficients of Basic Variables for Each Category (Top 2 Items)

	**PSH**			**Mental Change**
	**Survey1**	**Survey2**	**Average**	**Survey1**
Ascriptive factors(*4*)	0.085(6.8%)	0.058(5.3%)	0.072(6.1%)	0.072(19.5%)
	[5]	[5]	[5]	[2]
Biological factors(*3*/*2*)	0.233(18.7%)	0.402(36.5%)	0.318(27.0%)	0.060(16.3%)
	[2]	[1]	[2]	[3]*1*
Natural and Cultural factors(*2*)	0.204(16.3%)	0.237(21.5%)	0.221(18.8%)	
	[3]	[3]	[3]	
Economic factors(3)	0.061(4.9%)	0.082(7.4%)	0.072(6.1%)	0.039(10.6%)
	[6]*1*	[4]	[5]	[5]*1*
Societal community factors(4)	0.448(35.9%)	0.281(25.5%)	0.365(31.0%)	0.140(37.9%)
	[1]	[2]	[1]	[1]
Political factors(4)	0.217(17.4%)	0.042(3.8%)	0.130(11.0%)	0.058(15.7%)
	[4]	[6]*1*	[4]	[4]*1*
Notes: See Table 4.				

### Appendix E.2. Logistic Regression Analysis: Total Value of the Partial Regression Coefficients of Basic Factors in Each Category (Top 2 Items)

	**Light feelings increased**			**Dark feelings increased**			**Increased anxiety**			**Increased depression**		
	**Survey1**	**Survey2**	**Average**	**Survey1**	**Survey2**	**Average**	**Survey1**	**Survey2**	**Average**	**Survey1**	**Survey2**	**Average**
Ascriptive factors(*4*)	0.035	1.221	0.628	0.728	0.303	0.516	0.392	0.435	0.414	0.315	0.527	0.421
											
Biological factors(*3*/*2*)	0.088 (18.3%)	0.096 (28.0%)	0.092 (22.3%)	0.111 (22.8%)	0.054 (15.0%)	0.083 (19.5%)	0.075 (19.9%)	0.047 (12.4%)	0.061 (16.1%)	0.060 (14.8%)	0.051 (14.5%)	0.056 (14.7%)
	[3]*1*	[3]*1*	[2]	[3]	[3]*1*	[3]	[4]*1*	[4]*1*	[4]	[4]*1*	[3]*1*	[4]
Natural and Cultural factors(*2*)	0.169 (35.1%)		0.085 (20.5%)								0.043 (12.3%)	0.022 (5.7%)
	[1]*1*		[3]								[5]*1*	[5]
Economic factors(*3*)	0.083 (17.3%)		0.042 (10.1%)	0.066 (13.6%)	0.051 (14.2%)	0.059 (13.8%)	0.125 (33.2%)	0.109 (28.8%)	0.117 (31.0%)	0.082 (20.2%)	0.045 (12.8%)	0.064 (16.8%)
	[4]*1*		[5]	[4]*1*	[4]*1*	[4]	[1]	[2]	[1]	[3]*1*	[4]*1*	[3]
Societal community factors(*4*)		0.114 (33.2%)	0.057 (13.8%)	0.153 (31.4%)	0.189 (52.6%)	0.171 (40.4%)	0.076 (20.2%)	0.120 (31.7%)	0.098 (25.9%)	0.116 (28.6%)	0.154 (43.9%)	0.135 (35.7%)
		[2]*1*	[4]	[2]	[1]	[1]	[3]*1*	[1]	[3]	[2]*2*	[1]*3*	[1]
Political factors(*4*)	0.141 (29.3%)	0.133 (38.8%)	0.137 (33.3%)	0.157 (32.2%)	0.065 (18.1%)	0.111 (26.2%)	0.101 (26.8%)	0.103 (27.2%)	0.102 (27.0%)	0.148 (36.5%)	0.058 (16.5%)	0.103 (27.2%)
	[2]*1*	[1]*1*	[1]	[1]	[2]*1*	[2]	[2]	[3]*1*	[2]	[1]*4*	[2]*1*	[2]
Notes: See Table 6.												

## Appendix F. Logistic Regression Analysis: Emotional Change and Basic Factors (Surveys 1 and 2)

		**Increased light feelings**		**Increased dark feelings**		**Increased anxiety**		**Increased depression**	
		**Survey1**	**Survey2**	**Survey1**	**Survey2**	**Survey1**	**Survey2**	**Survey1**	**Survey2**
	Cox-Snell R2	0.019	0.013	0.046	0.038	0.062	0.053	0.064	0.069
	Nagelkerke R2	0.081	0.047	0.073	0.058	0.088	0.074	0.101	0.100
		***β***							
Ascriptive factors(4)	Sex		−0.606**	0.190*	0.299**	0.385**	0.427**	0.315**	0.321**
							
	Age	−0.035**		−0.008**	−0.004†	0.007**	0.008**		−0.019**
							
	Occupation		−0.615**	0.538**					−0.206*
							
	Marital status								
							
Biological factors(*3*/*2*)	Exercise/Foods	-	0.096*	-	−0.054**	-	−0.047**	-	−0.051**
		-	[3]	-	[4]	-	[5]	-	[5]
	Exercise		-		-	−0.075**	-	−0.060**	-
			-		-	[3]	-	[6]	-
	Foods	0.088†	-	−0.041†	-		-		-
		[3]	-	[7]	-		-		-
	Medical environment			−0.070**					
				[3]					
Natural and Cultural factors(*2*)	Natural environment						0.044*		
							[9]		
	Educational environment	0.169*					0.039†		−0.043†
		[1]					[8]		[7]
Economic factors(*3*)	Income			−0.066**	−0.051**		−0.069**		
				[5]	[5]		[3]		
	Assets					−0.049*	−0.040†		−0.045*
						[4]	[6]		[6]
	Employment stability	0.083†				−0.076**		−0.082**	
		[4]				[1]		[1]	
Societal community factors(*4*)	Stratification satisfaction		0.114**	−0.052*	−0.086**		−0.050**		−0.098**
			[2]	[6]	[2]		[4]		[1]
	General trust							−0.052*	
								[7]	
	Disparity recognition			0.101**	0.103**	0.076**	0.070**	0.064**	0.056**
				[1]	[1]	[1]	[2]	[4]	[3]
	Disparity elimination								−0.056**
									[3]
Political factors(*4*)	Fairness/Justice							−0.070*	
								[3]	
	Anti−corruptive fairness	−0.181**		−0.069**	−0.065**		−0.103**	−0.078**	
		[5]		[4]	[3]		[1]	[2]	
	Human rights	0.141†				0.058**		0.062**	−0.071**
		[2]				[6]		[8]	[2]
	Civil efficiency		0.133**	−0.088**	0.045*	−0.043**	0.031†	−0.063*	0.058**
			[1]	[2]	[6]	[5]	[7]	[5]	[8]
Notes: ** *p* < 0.01, * *p* < 0.05, † < 0.1 Independent variables: all basic factors, including ascriptive factors. PSH and notes of other variables: see Table 1, Appendix D.									

## Appendix G. Logistic Regression Analysis: Emotional Change and Factors Including Additional Fairness/Justice Items

		**Increased light feelings**	**Increased Dark feelings**	**Increased anxiety**	**Increased depression**
	Cox-Snell R2	0.013	0.038	0.055	0.070
	Nagelkerke R2	0.047	0.058	0.075	0.102
		***β***			
Ascriptive factors(4)	Sex	−0.606**	0.299**	0.430**	0.305**
			
	Age		−0.004†	0.008**	−0.020**
			
	Occupation	−0.615**			−0.214*
			
	Marital status				
			
Biological factors(*3*/*2*)	Exercise/foods	0.096*	−0.054**	−0.048**	−0.056**
		[3]	[4]	[6]	[4]
Natural and Cultural factors(*2*)	Natural environment			0.045*	
				[10]	
	Educational environment			0.041†	
				[9]	
Economic factors(*3*)	Income		−0.051**	−0.084**	
			[5]	[2]	
	Assets				−0.044*
					[6]
Societal community factors(*4*)	Stratification Satisfaction	0.114**	−0.086**	−0.053**	−0.101**
		[2]	[2]	[4]	[1]
	Disparity recognition		0.103**	0.053**	0.031**
			[1]	[4]	[7]
Political factors(*4*)	Anti-corruption fairness		−0.065**	−0.087**	
			[3]	[1]	
	Human rights				−0.046*
					[5]
	Civil Efficacy	0.133**	0.045*	0.030†	0.049**
		[1]	[6]	[8]	[8]
	Distributive Justice			−0.069**	−0.087**
				[3]	[2]
	Fair/Just Society			−0.044†	−0.058*
				[7]	[3]
Notes: ** *p* < 0.01, * *p* < 0.05, † *p* < 0.1. Independent variables: all basic factors, including Ascriptive factors, Distributive Justice, and Fair/Just society. Notes of other variables: see Table 1, Appendix D.					

## Appendix H. Multiple Regression Analysis of All Factors, Including All the Additional Ones (Survey 2)

		**PSH**
	R	0.882
	R2	0.778
	adjusted R2	0.778
		*β*
Ascriptive factors(*4*)	Sex	0.021**
		[12]
	Age	

	Occupation	0.021**
		[12]
	Marital Status	0.031**
		[10]
Biological factors(*3*/*2*)	Exercise/foods	0.181**
		[2]
	Medical environment	0.070**
		[7]
Natural and Cultural factors(*2*)	Natural environment	0.036**
		[9]
	Educational environment	0.063**
		[8]
Economic factors(*3*)	Income	0.025**
		[11]
	Assets	

	Employment stability	

Societal community factors(*4*)	Stratification satisfaction	0.095**
		[5]
	General trust	0.082**
		[6]
	Disparity recognition	

	Disparity elimination	

Political factors(*4*)	Fairness/Justice	

	Anti-corruptive fairness	−0.041**
		[15]
	Human rights	

	Civil efficiency	

	Hedonic	0.120**
		[4]
	Eudaimonic	0.177**
		[3]
	Contribution	

	Optimism	0.268**
		[1]
	Fair/Just Society	0.016**
		[14]
Notes: ** *p* < 0.01, Independent variables: all basic factors, including Ascriptive factors, Hedonic, Eudaimonic, Contribution, Optimism, and Fair/Just society. PSH and notes of other variables: see Table 1, Appendix D.		

## Appendix I. Logistic Regression Analysis of All Factors, Including All the Additional Ones (Survey 1 and 2)

		**Light feelings increased**		**Dark feelings increased**		**Increased anxiety**		**Increased depression**	
		**Survey1**	**Survey2**	**Survey1**	**Survey2**	**Survey1**	**Survey2**	**Survey1**	**Survey2**
	Cox-Snell R2	0.019	0.020	0.049	0.059	0.063	0.074	0.075	0.089
	Nagelkerke R2	0.079	0.074	0.078	0.090	0.089	0.102	0.118	0.130
		***β***							
Ascriptive factors(4)	Sex		−0.641**	0.206**	0.295**	0.394**	0.408**	0.372**	0.315**
							
	Age	−0.321**		−0.083**	−0.004†	0.057**	0.007**		−0.020**
							
	Occupation		−0.658**	0.577**					
							
	Marital status								
							
Biological factors(*3*/*2*)	Exercise/foods								0.037†
									[10]
	Exercise					−0.063**		−0.038*	
						[5]		[8]	
	Foods	0.087†							
		[3]							
	Medical environment			−0.046*					
				[6]					
Natural and Cultural factors(*2*)	Natural environment		−0.088†	0.082**		0.046*	0.047*	0.091**	
			[5]	[2]		[7]	[8]	[4]	
	Educational environment	0.168**							
		[1]							
Economic factors(*3*)	Income			−0.062**			−0.069**		
				[4]			[6]		
	Assets					−0.040*			
						[8]			
	Employment stability	0.084†				−0.072**		−0.068**	
		[4]				[3]		[7]	
Societal community factors(*4*)	Stratification satisfaction				−0.063**		−0.039*		−0.056**
					[8]		[9]		[6]
	General trust				0.043†				
					[9]				
	Disparity recognition		−0.068†		0.081**		0.033*		0.034†
			[6]		[4]		[10]		[11]
Political factors(*4*)	Fairness/Justice					−0.051†		−0.079*	
						[6]		[6]	
	Anti-corruptive fairness	−0.181**		−0.092**	−0.064**	−0.070**	−0.079**	−0.106**	
		[5]		[1]	[7]	[4]	[5]	[2]	
	Human rights	0.140**				0.078**		0.090**	−0.039†
		[2]				[2]		[5]	[9]
	Civil efficiency		0.137**	−0.055*					0.057**
			[3]	[5]					[12]
	Physical health				−0.072**	−0.569**	−0.119**	−0.103**	−0.097**
					[6]	[1]	[3]	[3]	[2]
	Mental health		0.268**	−0.072**	−0.235**			−0.184**	−0.238**
			[1]	[3]	[1]			[1]	[1]
	Hedonic		0.127†		0.131**		0.126**		0.074**
			[7]		[3]		[2]		[4]
	Contribution		−0.136**		0.149**		0.148**		0.083**
			[4]		[2]		[1]		[3]
	Optimism		0.143**		−0.074**		−0.118**		−0.071**
			[2]		[5]		[4]		[5]
	Distributive Justice						−0.062*		−0.054†
							[7]		[7]
	Fair/Just Society								−0.054†
									[7]
Notes: ** *p* < 0.01, * *p* < 0.05, † *p* < 0.1, Independent variables: all basic factors, including Ascriptive factors, Physical health, Mental health, Hedonic, Contribution, Optimism, Distributive Justice, and Fair/Just society. Notes of other variables: see Table 1, Appendix D.									
